# Supplementary material for: Segmenting functional tissue units across human organs using community-driven development of generalizable machine learning algorithms
Source: Nat Commun. 2023 Aug 3;14:4656. doi: 10.1038/s41467-023-40291-0 (PMC10400613; doi:10.1038/s41467-023-40291-0)
Supplement: Supplementary file 1 — Supplementary Information [file 41467_2023_40291_MOESM1_ESM.pdf]

Supplementary Information for Segmenting functional tissue units across human organs using community-driven development of generalizable machine learning algorithms

Authors

Yashvardhan Jain[1]*, Leah L. Godwin[1], Sripad Joshi[1], Shriya Mandarapu[1], Trang Le[2,3], Cecilia Lindskog[4], Emma Lundberg[2,3,5,6], Katy Börner[1]*

[1] Department of Intelligent Systems Engineering, Luddy School of Informatics, Computing, and Engineering, Indiana University, Bloomington, IN 47408, USA

[2] Science for Life Laboratory, School of Engineering Sciences in Chemistry, Biotechnology and Health, KTH - Royal Institute of Technology, Stockholm, Sweden

[3] Department of Bioengineering, Stanford University, Stanford, CA 94305, USA

[4] Department of Immunology, Genetics and Pathology, Division of Cancer Precision Medicine, Uppsala University, Uppsala, Sweden

[5] Department of Pathology, Stanford University, Stanford, CA 94305, USA

[6] Chan Zuckerberg Biohub, San Francisco, CA 94305, USA

*Corresponding authors

Yashvardhan Jain (yashjain@iu.edu)

Katy Börner (katy@indiana.edu)

Supplementary Notes

Supplementary Note 1. Team 1 - Extended Summary

1.1 Model Details

The final solution submitted by this team was an ensemble of 6 SegFormer[1] models (pretrained on CItyscapes[2] dataset):

- 1 x SegFormer mit-b3; image_size: 1024 x 1024
- 2 x SegFormer mit-b4; image_size: 960 x 960
- 1 x SegFormer mit-b5; image_size: 928 x 928
- 2 x SegFormer mit-b5; image_size: 960 x 960

All models used the implementations from the open-source MMSegmentation[3] library. The models used a weighted combination of Cross-entropy loss[4] (weight =1) and Lovasz loss[5] (weight = 3). AdamW[6] optimizer with a learning rate of 0.00006 was used and trained for 45000 steps. Training was done using a batch size of 1, with group normalization (number of groups = 32) for the decoder as it enabled training of the mit-b4 model with fewer resources and led to better results.

During training, several image augmentation techniques such as cropping, flipping, random rotate (90 degrees), HueSaturationValue, Random Gamma, and Random Brightness were implemented. Using mmSegmentation, the input images were resized to (480~1600, 480~1600), for example (480, 1600), (1024, 1024), (1500, 480). Random Crop was used with a crop_size = model_input size.

During inference, the pixel size of the images was ignored. Test-time augmentations (TTA) such as horizontal flip and scaling (approx. x0.8, x1.0, x1.2) were implemented. The prediction thresholds for each organ were defined as: 0.3 for kidney, 0.2 for large intestine, 0.6 for lung, 0.3 for prostate and 0.5 for spleen, after experimentation. Final prediction output was the average of sigmoid outputs across all models in the ensemble.

The team defined the problem as a 2-class problem (background and FTU) instead of a 6-class problem (background and 5 different organ classes) since this showed better results consistently, especially in the lungs. In the 6-class setup, the team found that the area of the lung tended to shrink and led to a worse leaderboard score for lung.

The team also found that while SegFormer mit-b5 was as good as mit-b4, the latter was more consistent in giving better results. It also gave the best result as an individual model (no ensemble) with a private LB score of 0.82821.

The team used Google Colaboratory (Pro +) with mostly Tesla V100 (16GB) and sometimes P100 (16GB) GPUs. They also, infrequently, used A100(40GB) GPU for some experiments but did not use that for the final submission models. Training cost about 4.5 hours for the SegFormer mit-b4 model using the Tesla V100.

1.2 External Datasets and Pseudo labeling

The team generated an additional dataset of 1,755 images from the 351 HPA training images by creating 4 additional pseudo-stained images (4 stain patterns) using the torchstain library. This allowed the team to create additional datasets that looked more like the H&E and PAS images in the test sets.

The team also downloaded 1 external spleen WSI and tiled it into 39 images. These were also converted into 39 x 4 stain patterns using the torchstain library. Additionally, the team downloaded 1 external lung WSI and tiled it into 73 images.

All external data used was pseudo-labeled by the team. For each organ in external data (spleen, lung), the team took the best performing model (at the time) and used that to generate the pseudo labels. These pseudo labels were then included as training data for the final models.

Additionally, the team also created pseudo labels for the given lung HPA data and dropped some noisy labels, and used these labels with a mix of given ground truth labels as training data for lung.

1.3 Ablation Study

Supplementary Table 1 provides the ablation study of various experiments conducted by the team. The experiments show SegFormer mit-b4 as the baseline model. Dealing with noisy lung labels and adding downscaled images for the prostate led to a combined boost of 0.13 on the private leaderboard. Adding various data augmentation and stain normalization techniques increased the score by another 0.05356. Other minor improvements came from adding external data and creating model ensembles. The table shows the mit-b4 single model performance in the bottom row and how scores improve with additional improvements.

Supplementary Note 2. Team 2 - Extended Summary

2.1 Model Details

The final solution submitted by this team was an ensemble of 4 U-Net style encoder-decoder models (3 CNN encoders and 1 transformer encoder) using the PyTorch Image Models[7] library:

- EfficientNet-b7[8]
- Convnext_large[9]
- Tf_efficientnetv2_l[10]
- Coat_lite_medium[11]

Coat performed the best as a single model but the ensemble with CNNs improved the score. The team also experimented with a few versions of Swin[12] v1 and v2 transformer models but found that these models gave worse performance.

All models were trained on 3 input sizes: 768x768, 1024x1024, and 1472x1472 with 5 folds. Additionally, the models were trained to predict the organ and pixel size as well. Adding these auxiliary tasks helped train the model to be more robust. The pixel size was calculated for resized input resolutions and changed during training augmentations.

The models used multiple loss functions: Dice+focal[13] loss for segmentation, Binary Cross Entropy (BCE) for organ classification, and Mean Squared Error (MSE) for pixel size prediction. Total loss was defined as: Dice_focal + 0.1 * bce + 0.1 * mse. It used Adan[14] optimizer with a learning rate of 0.0001 and used the ReduceLROnPlateau scheduler strategy. The models used half precision (float16) to reduce GPU memory. Batch size between 3-8 was used, depending on the encoder and resolution.

The training used heavy augmentation techniques such as random cropping/padding, scaling, rotating, flipping, color changing, blur/noise, saturation/brightness/contrast, elastic transform. During validation, test time augmentations such as flip, crop and padding were used. External data was separated on folds and used as validation to get best checkpoints. 4 test-time augmentations were used such as flip * 2 rotations to 90 degrees.

The prediction thresholds for each organ were defined as: 0.3 for kidney, 0.3 for large intestine, 0.06 for lung, 0.4 for prostate and 0.4 for spleen after experimentation. The final prediction is done using a weighted average across the 3 resolutions with weightage defined as 0.2 for 768x768, 0.3 for 1024x1024, and 0.5 for 1472x1472.

The models were trained using 2 NVIDIA RTX A6000 (48GB) GPUs. It took nearly 1 week of training time for the final ensemble. Inference took ~7-8 hours for final submission. For faster inference, the team suggested using a single coat_lite model with 768x768 resolution and no test-time augmentations.

2.2 External Datasets and Pseudo Labeling

The team used external data from previous competitions such as the "HuBMAP - Hacking the Human Kidney" competition[15], PANDA[16] challenge, and from Cancer Imaging Archive[17] (https://pathdb.cancerimagingarchive.net/imagesearch). A total of 80 images across these were used.

Additionally, the team downloaded 2,563 external HPA images from the Human Protein Atlas across all 5 organs.

All external data was pseudo labeled using the ensemble of initial models. The team also generated pseudo-labels for the given training data and used these pseudo-labels as ground truth for training (along with the original given ground truth) with a 30% probability.

The team used the staintools[18] library (https://hackmd.io/@peter554/staintools) using the method by Vahadane et al.[19] for stain normalization and generated additional pseudo-stained

images. For each image in the training set, 3 additional images were generated using this method. These new images were used with a 15% chance instead of originals during training.

2.3 Ablation Study

Since ablation study was not part of the submission requirements, not all teams kept track of their experiments. Hence, this team's ablation study is not presented.

Supplementary Note 3. Team 3 - Extended Summary

3.1 Model Details

The final solution submitted by this team[20] was an ensemble of 4 models:

- Unet++[21] with EfficientNet-b7 encoder
- Unet[22] with SegFormer mit-b5[23] encoder (Imagenet[24] pretrained)
- Unet with EfficientNet-b7 encoder. PointRend[25] was used as an additional loss for regularization, only for training.
- Unet with EfficientNet-b7 encoder. PointRend was used as an additional loss for regularization, only for training.

This ensemble achieved a private LB score of 0.83266.

The team also created another ensemble with a better score (0.83419) but did not select it as the final submission:

- Unet++ with EfficientNet-b7 encoder.
- Unet with SegFormer mit-b5 encoder. (Imagenet pretrained)
- Unet with SegFormer mit-b3 encoder. (Imagenet pretrained)

All models in the ensemble were trained for 5 folds and the final submission was averaged over all outputs. The team used implementations from the Segmentation Models Pytorch[26] (SMP) library.

During training, the team used a single channel output with half-precision (float16). They used the Adam[27] optimizer and a learning rate of 0.001, along with a reduceLRonPlateau strategy (patience=3, factor=0.5, min_lr=1e-7). The CNN models were trained using an image crop of 512x512 while the Mix Vision Transformer models used an image crop of 1024x1024. The total loss function was a combination of multiple losses: BCE, Dice, Focal[13] loss, Jaccard[28] Loss. The models used a batch size of 32.

The solution used heavy color augmentations to bridge the gap between varying color space between HPA and HuBMAP data such as: histogram matching[29], hue-value-saturation, contrast, gamma augmentations. Additional geometric augmentations such as: random flips, rotations, scales, shifts, elastic transforms. In some experiments, the team tried stain normalization techniques used by other teams but did not see much improvement, perhaps due to the already applied color augmentations.

The team noticed a major improvement by using the CutMix[30] augmentation. This was applied with a probability of 0.5 and patches were sampled randomly. This was applied for images within a single class for each class. CutMix samples a random part of an image and replaces it with a patch from another image.

During inference, the team averaged the 3 best checkpoints for each fold. They used 3 flips as test-time augmentation. The final masks were processed to remove small regions: regionArea/ImageArea < OrganThresh, where organThresh is: 0.001 (kidney), 0.0005

(prostate), 0.0001 (large intestine), 0.001 (spleen) and 0.000001 (lung). The threshold values were defined after experimenting with different values. The team used a 1024x1024 sliding window approach with 0.75 overlap for the transformer models (for CUDA memory reasons) while predictions were on full scale images for CNN models.

Training was done using the A100 GPUs.

3.2 External Datasets and Pseudo Labeling

The team created an additional HPA dataset by rescaling all images to match the pixel size of HuBMAP images for all organs to adapt to varying image resolutions.

The team also downloaded 140 images from GTEx (https://gtexportal.org/home) for prostate, kidney, large intestine, and spleen, for patients with no apparent pathologies. These were progressively added to the pipeline for pseudo labeling. Additionally, the team also downloaded between 57,000 and 61,000 images from HPA for each organ.

Pseudo labeling was done with the best ensemble without pseudo labels. They did not use the entire HPA pseudo labeled data for each epoch, but randomly sampled from the large dataset. The pseudo labeling was repeated twice.

All external data (in total, 422 GB of data for HPA+GTEx, 6GB for labels) was made publicly available by the team as Kaggle Datasets:

- https://www.kaggle.com/datasets/igorkrashenyi/liver-hpa-pt0
- https://www.kaggle.com/datasets/igorkrashenyi/liver-hpa-pt2
- https://www.kaggle.com/datasets/igorkrashenyi/liver-hpa-pt1
- https://www.kaggle.com/datasets/igorkrashenyi/hap-kidney-dataset-pt1

- https://www.kaggle.com/datasets/igorkrashenyi/kidney-hpa-dataset-pt0
- https://www.kaggle.com/datasets/igorkrashenyi/hpa-colon-dataset
- https://www.kaggle.com/datasets/igorkrashenyi/hpa-spleen-dataset-pt1
- https://www.kaggle.com/datasets/igorkrashenyi/hpa-spleen-dataset-pt0
- https://www.kaggle.com/datasets/igorkrashenyi/hpa-prostate-dataset
- https://www.kaggle.com/datasets/igorkrashenyi/lung-hpa-dataset
- https://www.kaggle.com/datasets/sakvaua/gtex-pseudo-humantorusteam (GTEx)
- https://www.kaggle.com/datasets/vladimirsydor/hubmap-2022-add-data-labels-v2 (all pseudo labels)

3.3 Ablation Study

Supplementary Table 2 shows the ablation study conducted by the team. We see that bridging the domain gap between the HPA and HuBMAP data via pixel size adaptation, histogram matching, and CutMix augmentation gives a major boost to the performance and takes the private score from 0.15632 to 0.64518. Second major improvement comes from adding external data and pseudo labels, with addition of GTEx data improving the score by 0.07165 and addition of HPA data improving the score by 0.0305. Lung sees the most significant improvement and goes from 0.04 to 0.48, while others see relatively smaller but significant improvements: 0.85 to 0.95 (kidney), 0.87 to 0.91 (large intestine), 0.81 to 0.83 (prostate), 0.69 to 0.84 (spleen). The table shows the best single model performance in the top row and how scores improve with additional improvements as we go down in the table.

Supplementary Note 4. Alveoli segmentations in lung tissue

Due to confusion regarding varied looking alveoli segmentations in lung tissue images, additional information was provided to the teams. The data included masks of both atelectatic (collapsed) and inflated alveoli (un-collapsed). The alveolar appearance on the image slides

depends on how the tissue samples were prepared. For the inflated alveoli, which have a 3D ‘cup’ shaped structure, how the tissue is sectioned can cause variability as well. If the alveoli were sectioned in a horizontal manner, their shape will appear more like a complete circle. Whereas if the alveoli were sectioned vertically, they may appear more as a U-shape.

Supplementary Note 5. Judges’ Prizes Rubric

5.1 Scientific Prize

Kaggle teams were asked to investigate correlations between predicted FTUs (e.g., area and shape) and donor demographics (e.g., sex and age). The evaluation rubric further emphasized validation of methods and implementations, documentation of performance and limitations, novelty of solutions, and presentation of insights useful for generation of reference FTUs for inclusion into a Human Reference Atlas. Scientific Prize winners were identified by a panel of human experts who selected two teams to receive equal Scientific Prize amounts ($10,000 each) based on the submission’s contribution to science and demonstration of innovative approaches. The complete evaluation rubric, as presented below, consisted of eight criteria which were used by the judges to evaluate the winners. Each criterion consisted of ten points for a total of 80 points.

1. Are the statistical and modeling methods used to identify FTUs appropriate for the task?
2. Are confidence scores and other metrics provided that help interpret the results achieved by the segmentation methods?
3. Is the presented characterization of FTUs useful for understanding individual differences, e.g., the impact of donor sex and age on the shape, size or spatial distribution of FTUs?
4. Is it possible to predict FTU area size distribution, given age and sex information across all organs?

5. Did the team validate their methods and algorithm implementations and provide information on algorithm performance and limitations?
6. Did the team document their method and code appropriately?
7. Did the team develop a creative or novel method to segment FTUs?
8. Did the team provide insights that would be useful for generating reference FTUs for inclusion into a Human Reference Atlas?

5.2 Diversity and Presentation Prize

The complete evaluation rubric, as presented below, consisted of three criteria which were used by the judges to evaluate the winner. Each criterion consisted of ten points for a total of 30 points.

1. Does the team embrace diversity and equity, welcoming team members of different ages, genders, ethnicities, and with multiple backgrounds and perspectives?
2. Did the authors effectively communicate the details of their method for segmenting FTUs, and the quality and limitations of their results? For example, did they use data visualizations to present algorithm setup, run, results and/or to provide insight into the comparative performance of different methods? Were these visualizations effective at communicating insights about their approach and results to experts and novice users?
3. Are the important results easily understood by the average person?

Supplementary Tables

Supplementary Table 1. Ablation Study by Team 1. This table lists the ablation study done by the winning team, detailing the strategies that helped improve the performance of their solution. Private Dice shows the mean Dice scores on the private test set. Public Dice shows the mean Dice scores on the public test set. Public HuBMAP Dice shows the mean Dice scores on only the HuBMAP data in the public test set (removing predictions for the HPA data in the public test set). Private Dice Gain shows the mean Dice score change for the current experiment relative to the row below. Baseline model shown in the bottommost row.

SUPPLEMENTARY TABLE 1: Ablation Study by Team 1

Short description*	Private Dice	Public Dice	Public HuBMAP Dice	Private Dice Gain
6 model ensemble	0.83562	0.82716		0.00094
5 model ensemble	0.83468	0.82679		0.00088
4 model ensemble + image ratio divisor 32	0.8338	0.82622		0.00142
3 model ensemble	0.83238	0.82364		0.00417
mit-b4_0.8_1_1.2 multiple scales	0.82821		0.60718	0.00035
mit-b4_optimized_threshold	0.82786		0.60663	0.00335
mit-b4 aug + brightness	0.82451		0.6046	0.00853
mit-b4 aug + HPA original (not stained)	0.81598	0.81661		-0.00456
mit-b4 + external lung	0.82054	0.81345	0.60057	0.00622
mit-b4 + external spleen	0.81432		0.59722	0.00594
mit-b4 + stain transfer (torchstain)	0.80838	0.80858	0.59243	0.00927
mit-b4 + pseudo label (prostate, intestine) patch	0.79911		0.58466	0.00112
mit-b4 + trainval (351 images)	0.79799	0.80006	0.58248	0.00481
mit-b4 + lung annotation	0.79318		0.58097	0.07256
mit-b4 + albu dataaug +pseudo label (prostate, intestine)	0.72062	0.76051	0.54628	0.02496
mit-b4 + better resize	0.69566		0.52913	0.0108
mit-b4 + prostate_downscale	0.68486		0.52633	0.05791
mit-b4 (baseline reference model)**	0.62695		0.47984	0

*List is non-exhaustive and does not include all experiments by the team. Only listed is the subset of experiments team tracked and provided.

**All experiments and performance gains are relative to this model.

Supplementary Table 2: Ablation Study by Team 3. This table lists the ablation study done by the team that won the third performance prize, detailing the strategies that helped improve the performance of their solution. Private Dice shows the mean Dice scores on the private test set. Public HuBMAP Dice shows the mean Dice scores on only the HuBMAP data in the public test set (removing predictions for the HPA data in the public test set). Out of Fold Dice shows mean Dice scores for the training data in other four folds (based on 5-fold validation). Private Dice Gain shows the mean Dice score change for the current experiment relative to the row above. Baseline model shown in the topmost row.

SUPPLEMENTARY TABLE 2: Ablation Study by Team 3

Short Description**	Out of Fold Dice	Public HuBMAP Dice*	Private Dice	Private Dice Gain
**Baseline model**	0.71172	0.12115	0.15632	-
+ Pixel size adaptation	0.71588	0.22653	0.30195	0.14563
+ Histogram matching	0.70283	0.38732	0.48486	0.18291
+ 1 output channel + CutMix	0.75157	0.49483	0.64518	0.16032
+ Heavy augmentations	0.7695	0.51506	0.71187	0.06669
+ Additional scalers + External GTEx data with pseudo labels	0.82142	0.58037	0.78352	0.07165
+ Additional HPA data with pseudo labels	0.83633	0.598	0.81402	0.0305
+ Best 5 folds solo model	0.85405	0.60826	0.83332	0.0193
+ Ensemble	0.85428	0.60931	0.83419	0.00087

* Public HuBMAP Dice involves setting predictions for public test HPA data to zero.

** List is non-exhaustive and does not include all experiments by the team. Only listed is the subset of experiments team tracked and provided.

Supplementary Table 3: Results for run time and accuracy from pilot run using baseline model. This table provides the run time and accuracy from the pilot run using the baseline model (winning solution from the previous competition).

Supplementary Table 3: Results for run time and accuracy from pilot run using baseline model

Algorithm	Training Time (seconds)	Inference Time (seconds)	Mean Dice (Private HPA)	Mean Dice (HuBMAP)	Mean Dice (Private HPA + HuBMAP)
Tom	18,339	1,424	0.76	0.53	0.57

Supplementary Table 4: Mean Dice Scores and Mean IOU Scores for Top-50 Teams. This table provides the mean Dice and mean IOU scores for the top-50 teams on the private test set. Rankings are based on the final private leaderboard scores at the end of the competition.

Supplementary Table 4: Mean Dice Scores and Mean IOU Scores for Top-50 Teams

Team Rank*	Mean Dice	Mean IOU
Team_1	0.835616	0.738462
Team_2	0.833929	0.736247
Team_3	0.832661	0.733348
Team_4	0.824744	0.724394
Team_5	0.825953	0.727103
Team_6	0.825355	0.727711
Team_7	0.825162	0.725917
Team_8	0.824804	0.724025
Team_9	0.823342	0.724083
Team_10	0.822665	0.722829
Team_11	0.820494	0.718804
Team_12	0.819907	0.720152
Team_13	0.81913	0.717076
Team_14	0.818784	0.717634
Team_15	0.817438	0.717222
Team_16	0.814089	0.713238
Team_17	0.814948	0.713123
Team_18	0.814632	0.713361
Team_19	0.814387	0.713701
Team_20	0.809155	0.702849
Team_21	0.80365	0.695938
Team_22	0.811637	0.71121
Team_23	0.81258	0.71089
Team_24	0.812443	0.710357
Team_25	0.812318	0.709355
Team_26	0.810068	0.700955
Team_27	0.808477	0.703046
Team_28	0.805709	0.698541
Team_29	0.796718	0.688948
Team_30	0.789057	0.685176
Team_31	0.802974	0.697731
Team_32	0.80669	0.705497
Team_33	0.800047	0.695258
Team_34	0.805063	0.699041
Team_35	0.805182	0.701083
Team_36	0.805535	0.699149
Team_37	0.798853	0.695286
Team_38	0.797459	0.694136
Team_39	0.802248	0.694805
Team_40	0.791561	0.68686
Team_41	0.781748	0.671092
Team_42	0.801776	0.697441
Team_43	0.801306	0.697886
Team_44	0.799077	0.69235
Team_45	0.79995	0.69615
Team_46	0.799475	0.69139
Team_47	0.799184	0.694519
Team_48	0.798772	0.691864
Team_49	0.786115	0.6777
Team_50	0.796606	0.690417

* Ranking based on the private leaderboard.

Supplementary Figures

Supplementary Figure 1. Tissue region percentage vs. number of images against different threshold values for public HPA data for all five organs. Final selected tissue region percentage thresholds: 5% for lung and 15% for other four organs. Source data are provided as a source data file on Zenodo (see data availability statement).

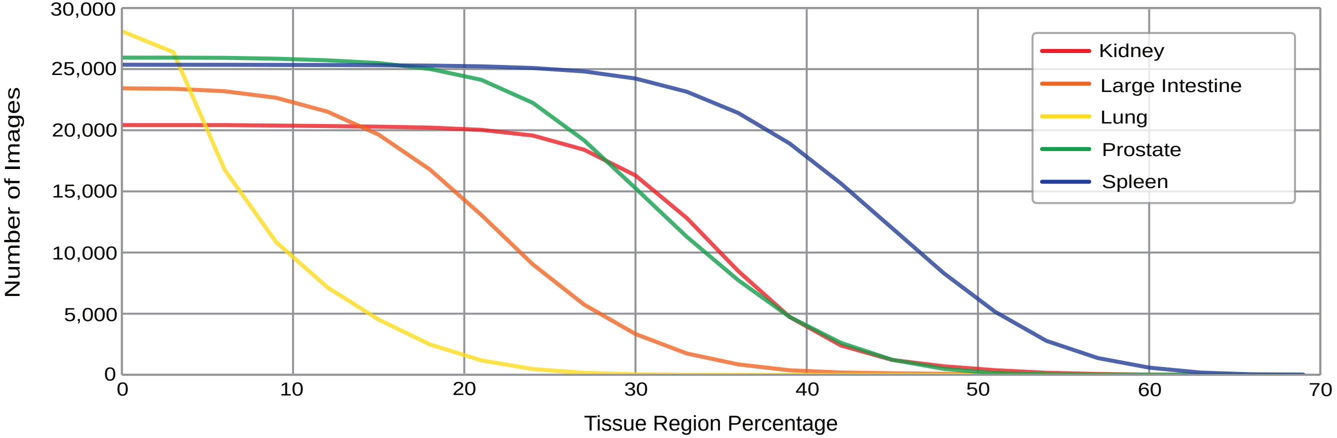
Number of Images
30,000
25,000
20,000
15,000
10,000
5,000
0
0
10
20
30
40
50
60
70
Tissue Region Percentage
Kidney
Large Intestine
Lung
Prostate
Spleen

Supplementary Figure 2. Tissue samples per organ and age for all 5 organs. a. Donor distribution color coded by male (blue) and female (red). **b.** Donor age and sex distribution color coded by HuBMAP (green) and HPA (purple). Source data are provided as a source data file on Zenodo (see data availability statement).

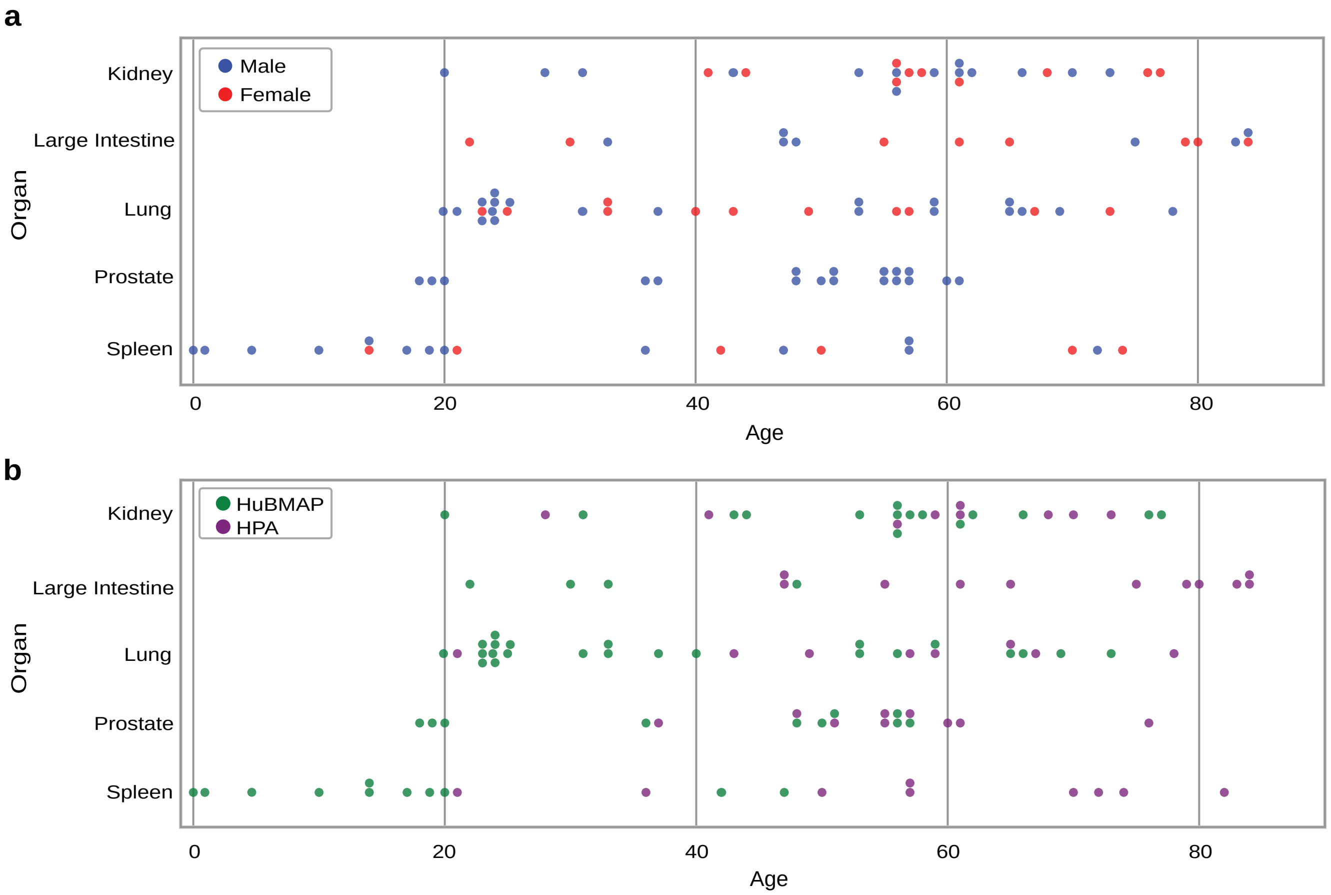

a
Male
Female
Kidney
Large Intestine
Lung
Prostate
Spleen
Organ
0
20
40
60
80
Age
b
HuBMAP
HPA
Kidney
Large Intestine
Lung
Prostate
Spleen
Organ
0
20
40
60
80
Age

Supplementary Figure 3. Best five predictions for kidney images from Team 1. Ground truth and predictions for Team 1’s final solution are shown, along with the prediction overlay and mask difference with per-pixel false positives (in blue) and false negatives (red).

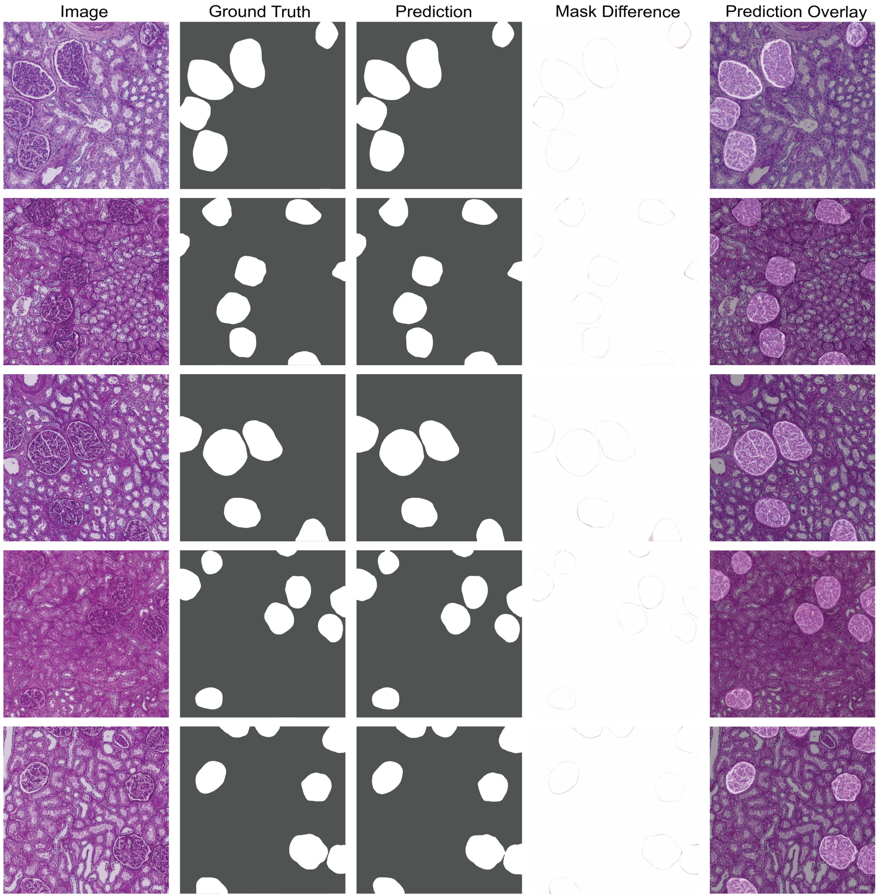
Image
Ground Truth
Prediction
Mask Difference
Prediction Overlay

Supplementary Figure 4. Worst five predictions for kidney images from Team 1. Ground truth and predictions for Team 1's final solution are shown, along with the prediction overlay and mask difference with per-pixel false positives (in blue) and false negatives (red).

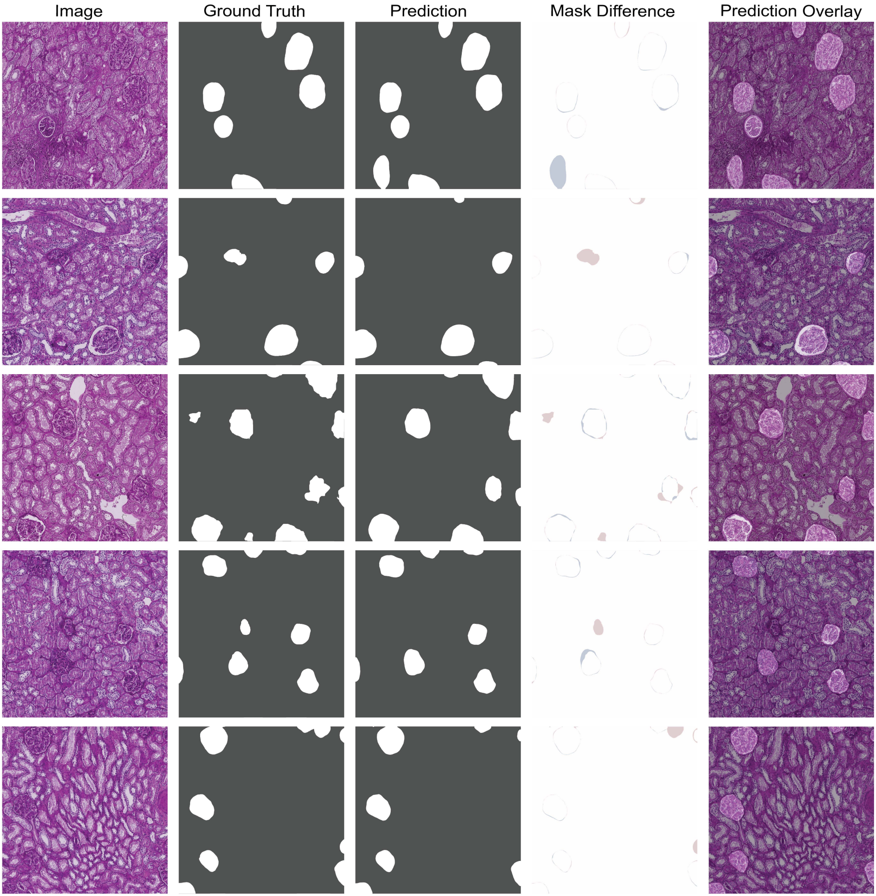
Image
Ground Truth
Prediction
Mask Difference
Prediction Overlay

Supplementary Figure 5. Best five predictions for large intestine images from Team 1. Ground truth and predictions for Team 1's final solution are shown, along with the prediction overlay and mask difference with per-pixel false positives (in blue) and false negatives (red).

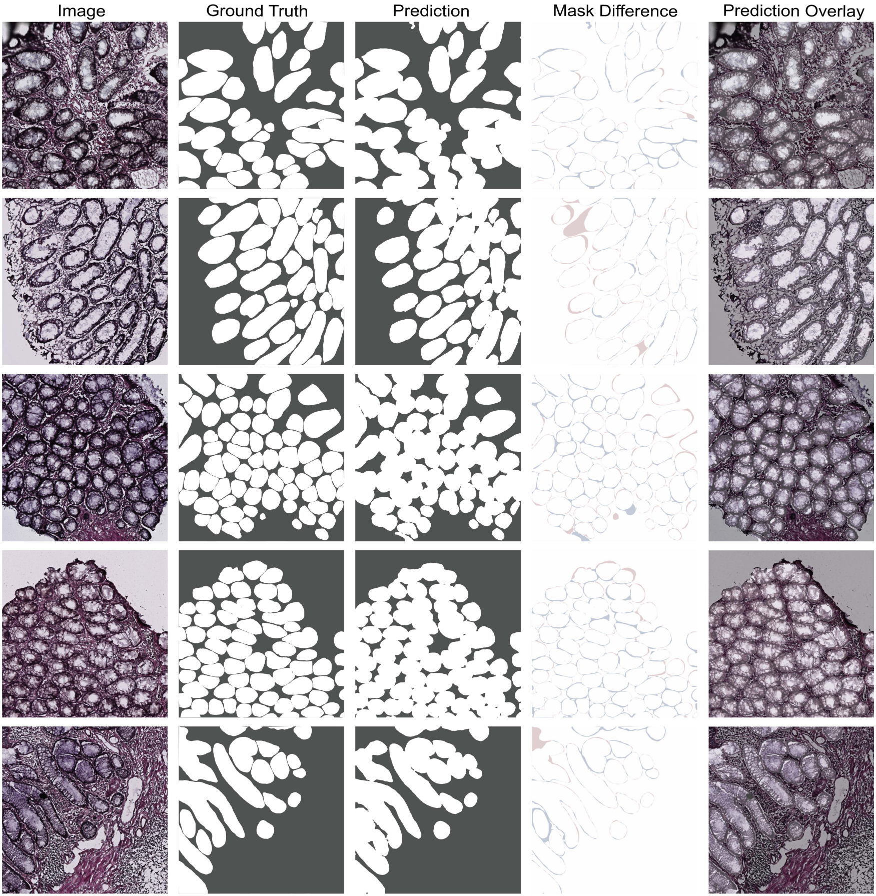
Image
Ground Truth
Prediction
Mask Difference
Prediction Overlay

Supplementary Figure 6. Worst five predictions for large intestine images from Team 1. Ground truth and predictions for Team 1's final solution are shown, along with the prediction overlay and mask difference with per-pixel false positives (in blue) and false negatives (red).

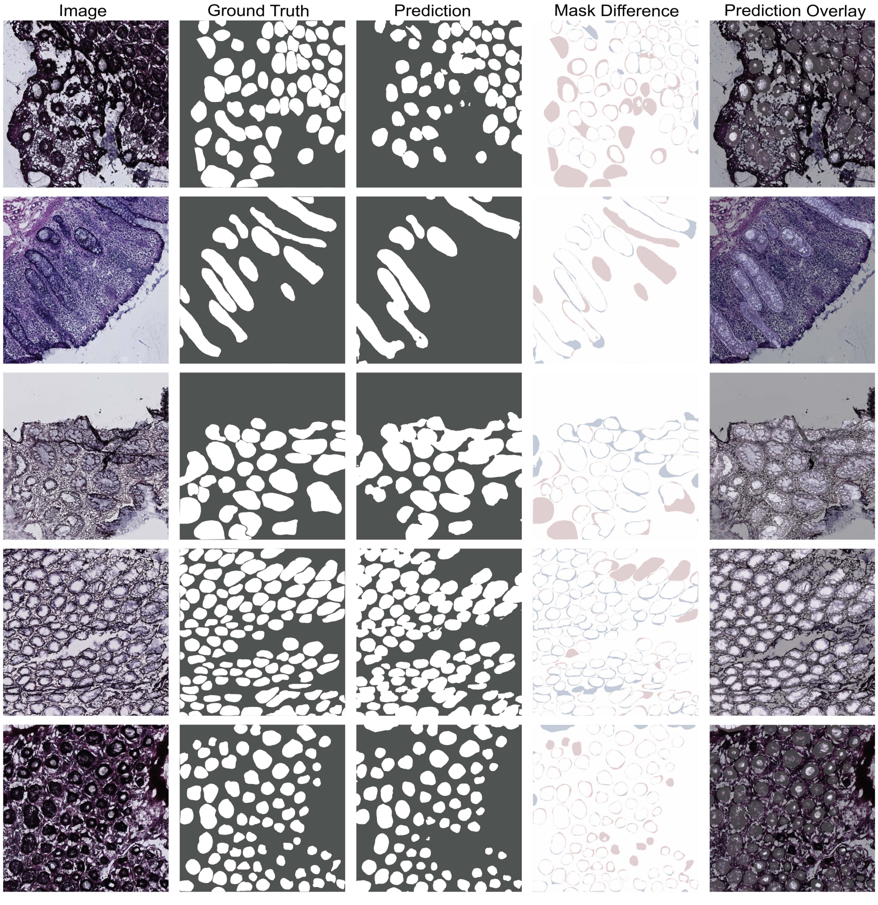
Image
Ground Truth
Prediction
Mask Difference
Prediction Overlay

Supplementary Figure 7. Best five predictions for lung images from Team 1. Ground truth and predictions for Team 1's final solution are shown, along with the prediction overlay and mask difference with per-pixel false positives (in blue) and false negatives (red).

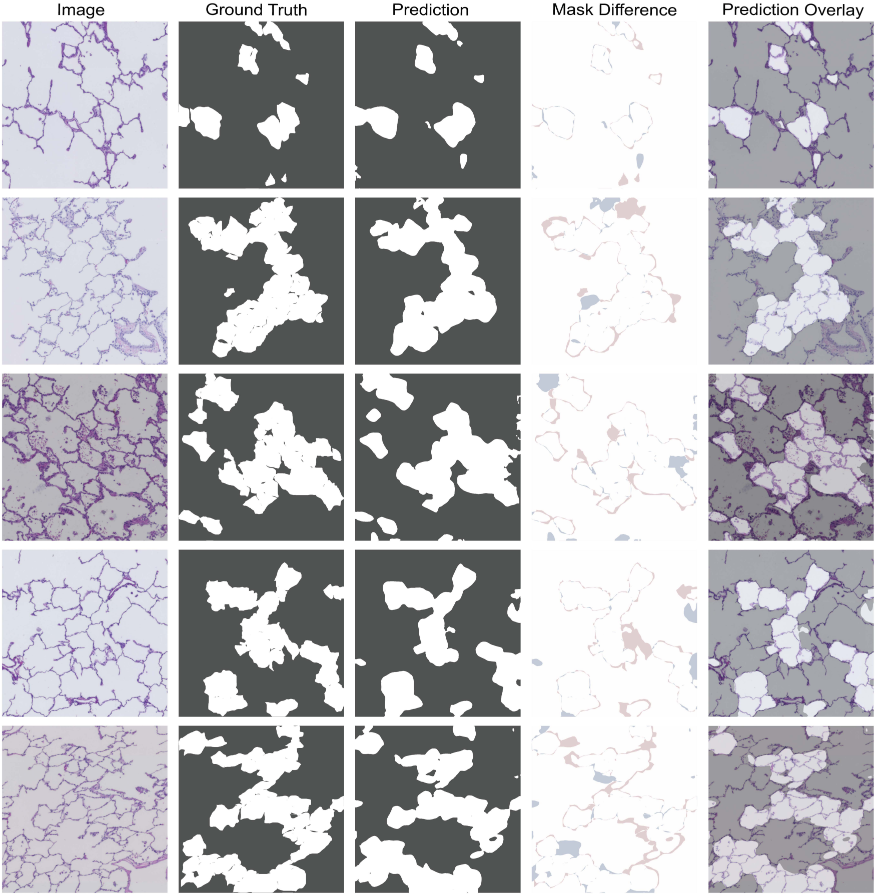
Image
Ground Truth
Prediction
Mask Difference
Prediction Overlay

Supplementary Figure 8. Worst five predictions for lung images from Team 1. Ground truth and predictions for Team 1's final solution are shown, along with the prediction overlay and mask difference with per-pixel false positives (in blue) and false negatives (red).

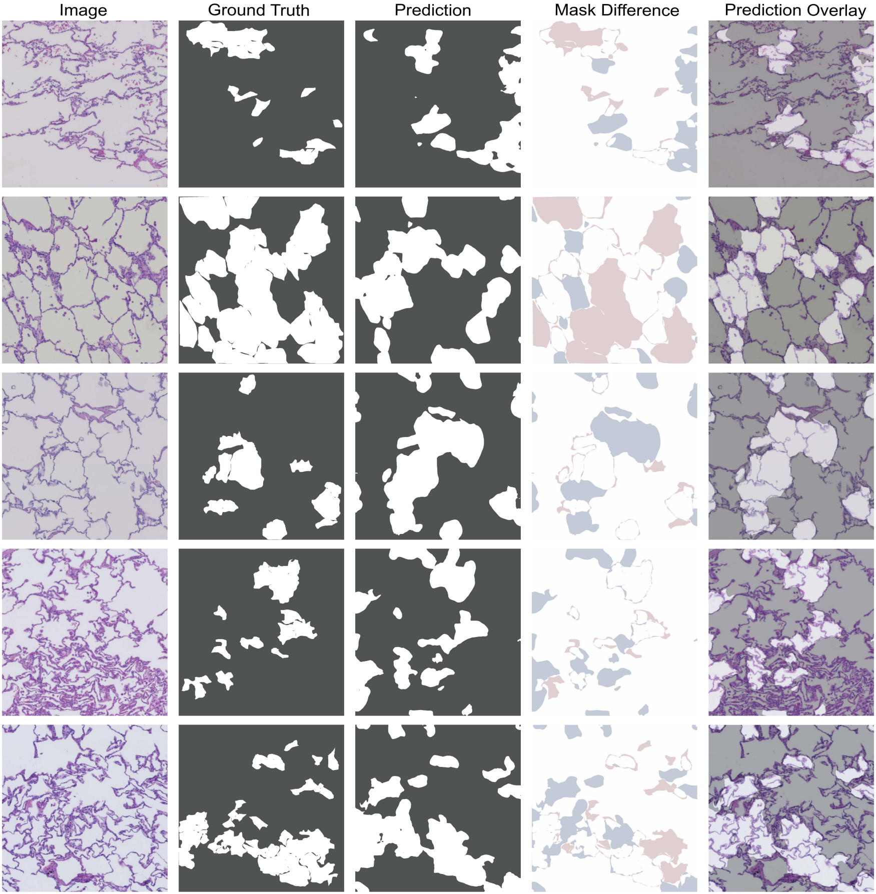
Image
Ground Truth
Prediction
Mask Difference
Prediction Overlay

Supplementary Figure 9. Best five predictions for prostate images from Team 1. Ground truth and predictions for Team 1's final solution are shown, along with the prediction overlay and mask difference with per-pixel false positives (in blue) and false negatives (red).

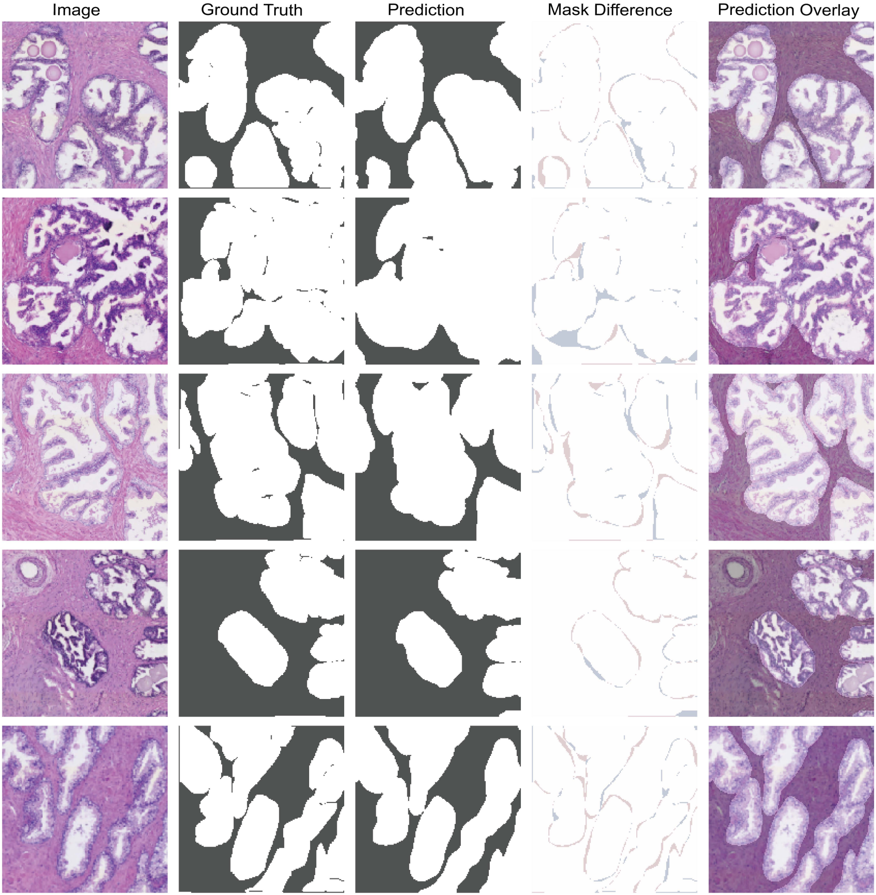
Image
Ground Truth
Prediction
Mask Difference
Prediction Overlay

Supplementary Figure 10. Worst five predictions for prostate images from Team 1. Ground truth and predictions for Team 1's final solution are shown, along with the prediction overlay and mask difference with per-pixel false positives (in blue) and false negatives (red).

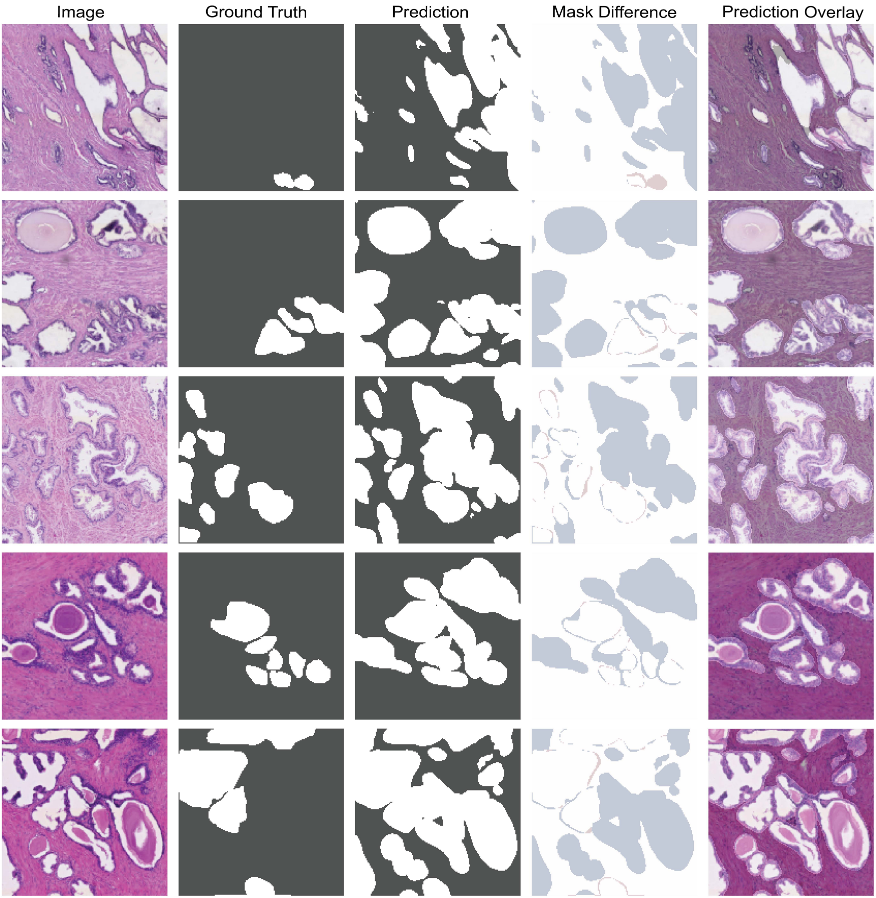
Image
Ground Truth
Prediction
Mask Difference
Prediction Overlay

Supplementary Figure 11. Best five predictions for spleen images from Team 1. Ground truth and predictions for Team 1's final solution are shown, along with the prediction overlay and mask difference with per-pixel false positives (in blue) and false negatives (red).

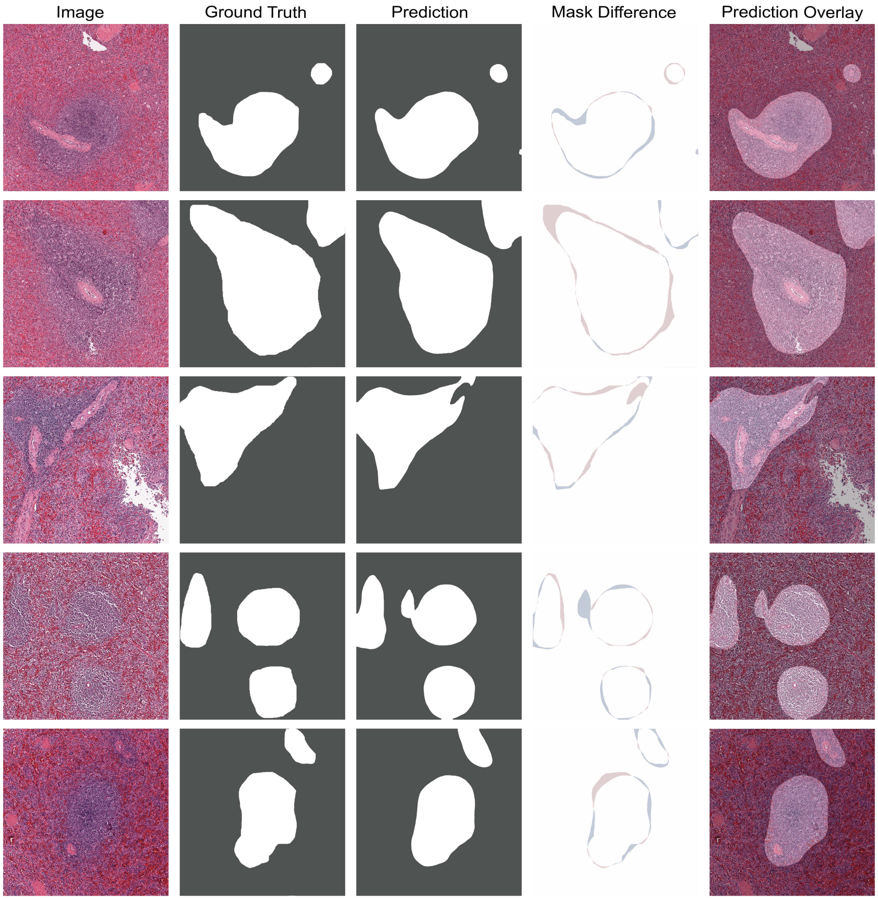
Image
Ground Truth
Prediction
Mask Difference
Prediction Overlay

Supplementary Figure 12. Worst five predictions for spleen images from Team 1. Ground truth and predictions for Team 1's final solution are shown, along with the prediction overlay and mask difference with per-pixel false positives (in blue) and false negatives (red).

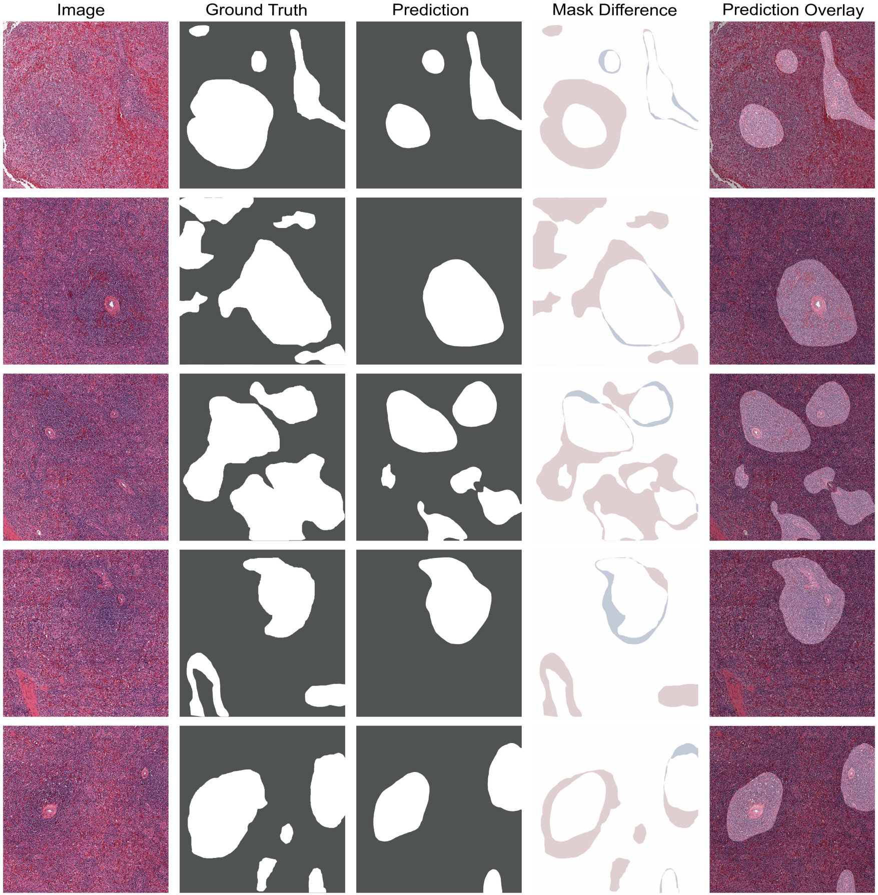

Image
Ground Truth
Prediction
Mask Difference
Prediction Overlay

Supplementary Figure 13. Best five predictions for kidney images from Team 2. Ground truth and predictions for Team 2's final solution are shown, along with the prediction overlay and mask difference with per-pixel false positives (in blue) and false negatives (red).

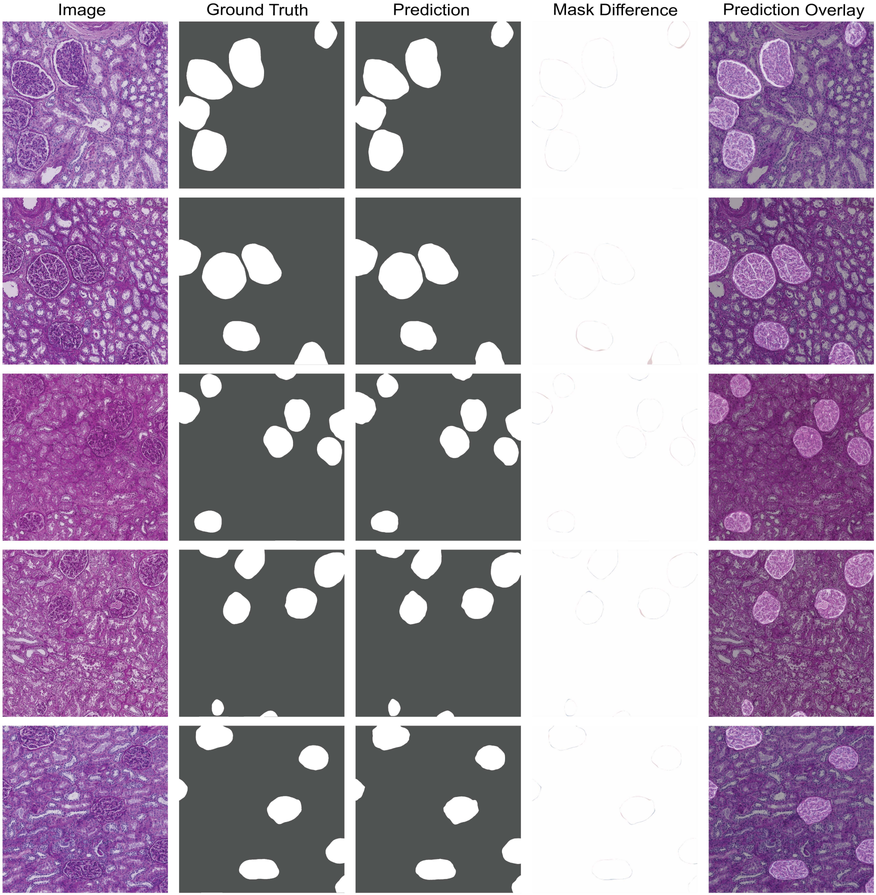
Image
Ground Truth
Prediction
Mask Difference
Prediction Overlay

Supplementary Figure 14. Worst five predictions for kidney images from Team 2. Ground truth and predictions for Team 2's final solution are shown, along with the prediction overlay and mask difference with per-pixel false positives (in blue) and false negatives (red).

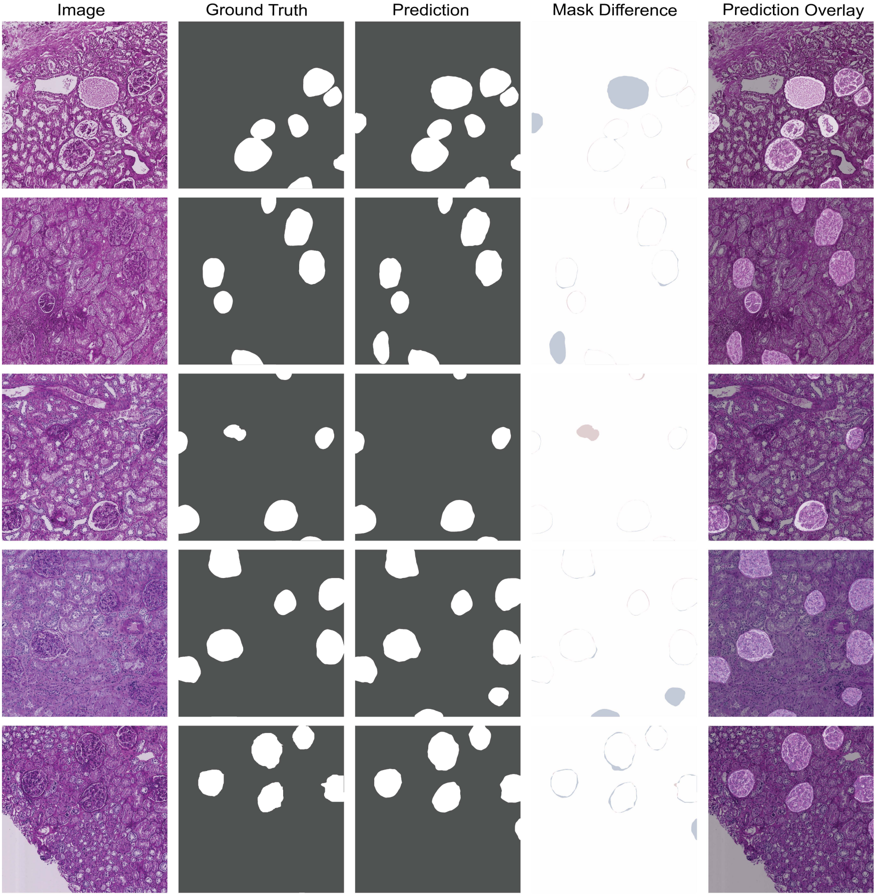

Image
Ground Truth
Prediction
Mask Difference
Prediction Overlay

Supplementary Figure 15. Best five predictions for large intestine images from Team 2. Ground truth and predictions for Team 2’s final solution are shown, along with the prediction overlay and mask difference with per-pixel false positives (in blue) and false negatives (red).

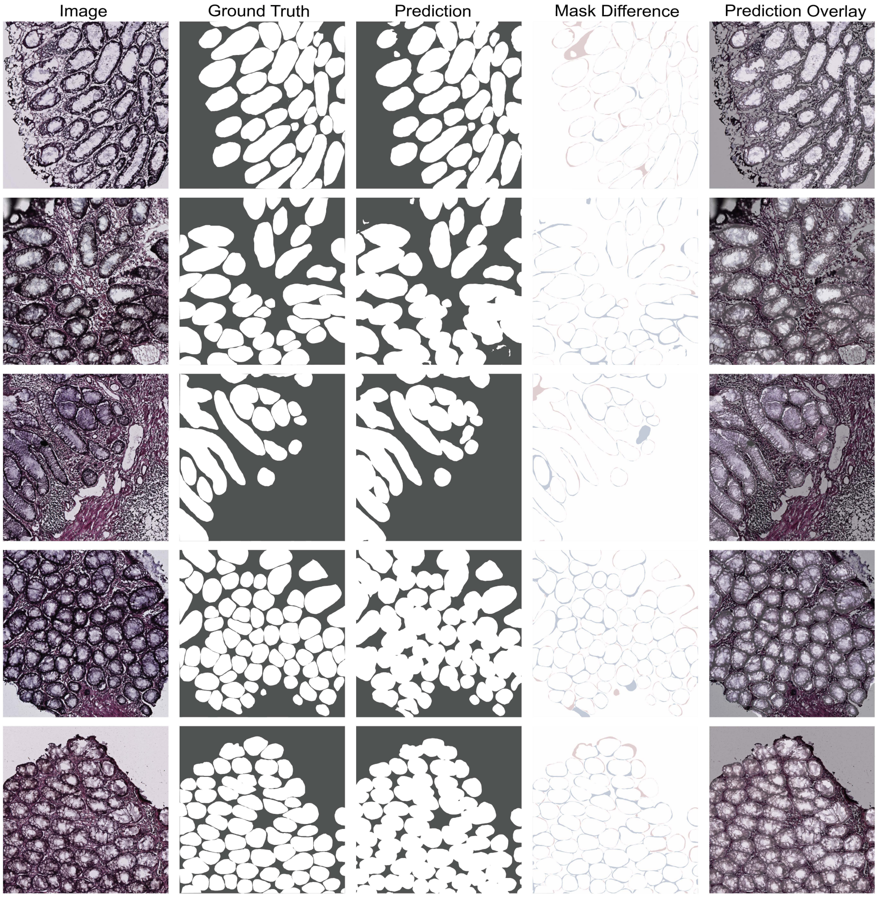
Image
Ground Truth
Prediction
Mask Difference
Prediction Overlay

Supplementary Figure 16. Worst five predictions for large intestine images from Team 2. Ground truth and predictions for Team 2's final solution are shown, along with the prediction overlay and mask difference with per-pixel false positives (in blue) and false negatives (red).

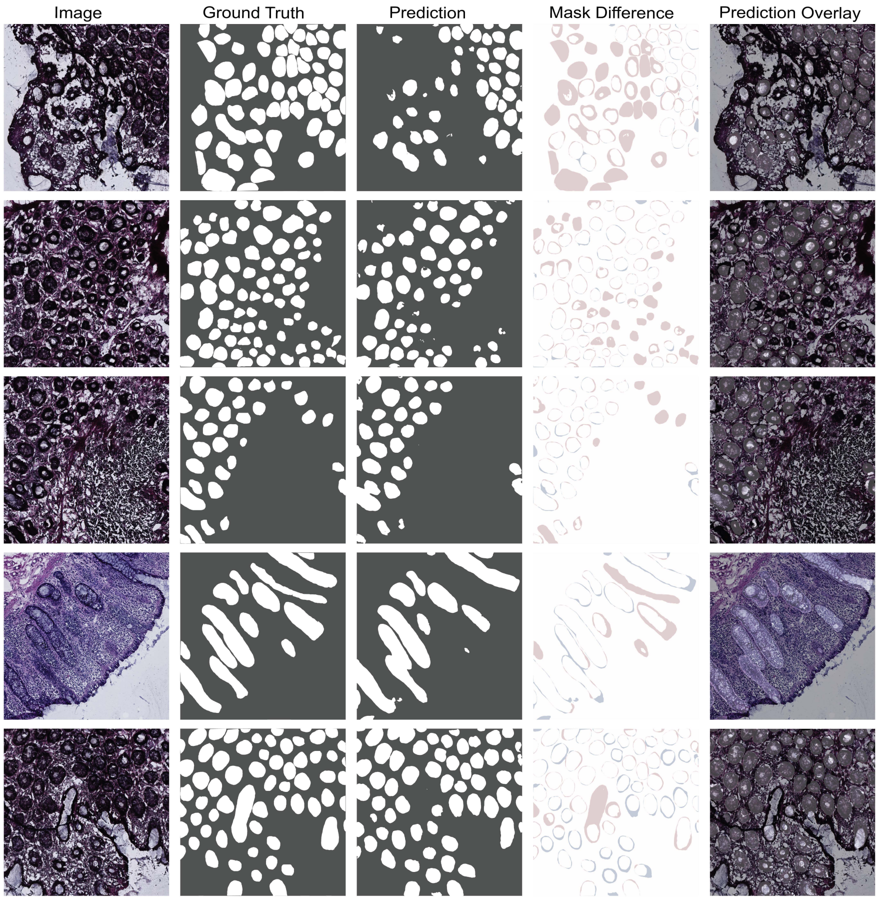
Image
Ground Truth
Prediction
Mask Difference
Prediction Overlay

Supplementary Figure 17. Best five predictions for lung images from Team 2. Ground truth and predictions for Team 2's final solution are shown, along with the prediction overlay and mask difference with per-pixel false positives (in blue) and false negatives (red).

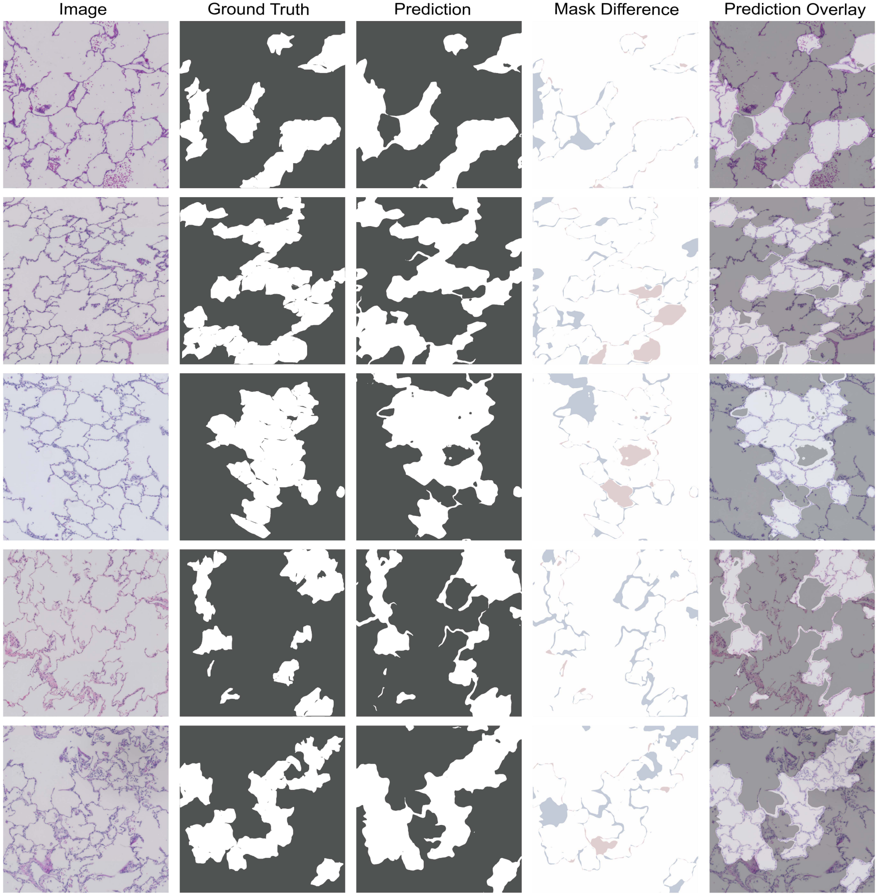

Image
Ground Truth
Prediction
Mask Difference
Prediction Overlay

Supplementary Figure 18. Worst five predictions for lung images from Team 2. Ground truth and predictions for Team 2's final solution are shown, along with the prediction overlay and mask difference with per-pixel false positives (in blue) and false negatives (red).

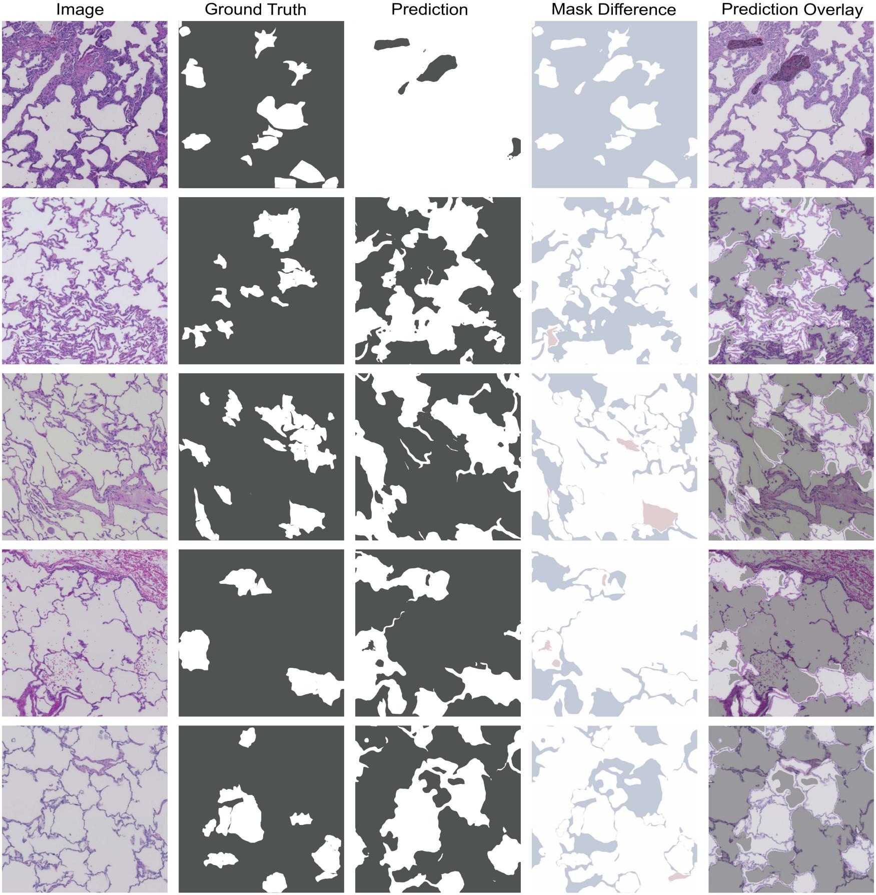
Image
Ground Truth
Prediction
Mask Difference
Prediction Overlay

Supplementary Figure 19. Best five predictions for prostate images from Team 2. Ground truth and predictions for Team 2’s final solution are shown, along with the prediction overlay and mask difference with per-pixel false positives (in blue) and false negatives (red).

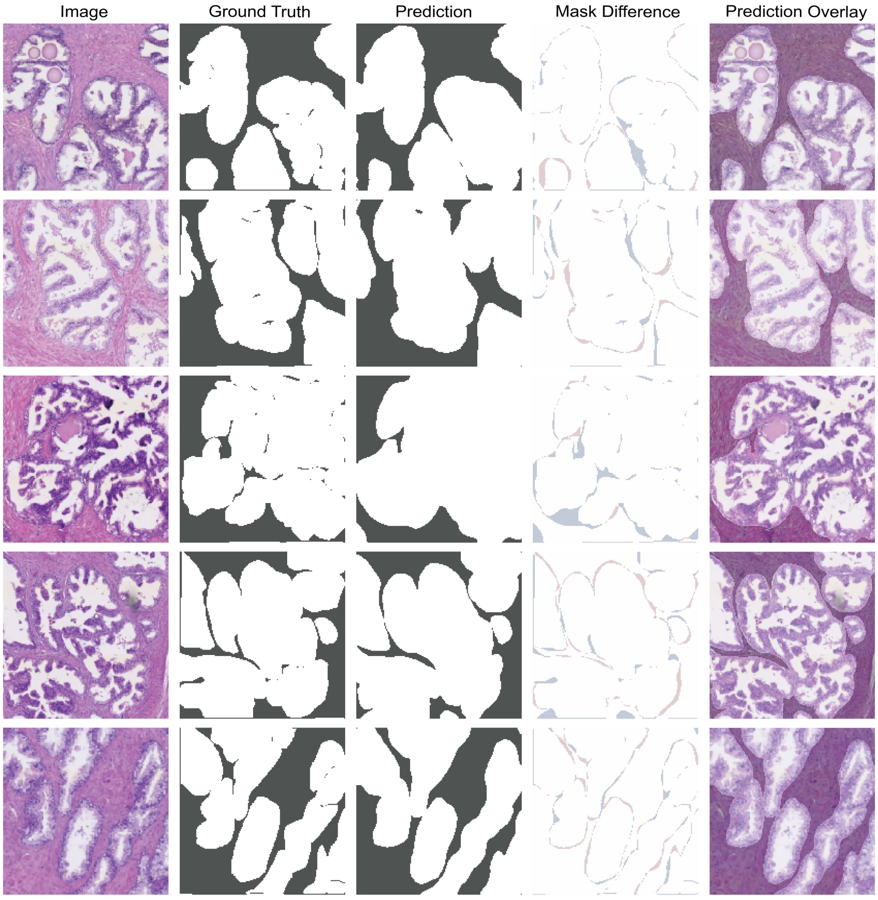
Image
Ground Truth
Prediction
Mask Difference
Prediction Overlay

Supplementary Figure 20. Worst five predictions for prostate images from Team 2. Ground truth and predictions for Team 2's final solution are shown, along with the prediction overlay and mask difference with per-pixel false positives (in blue) and false negatives (red).

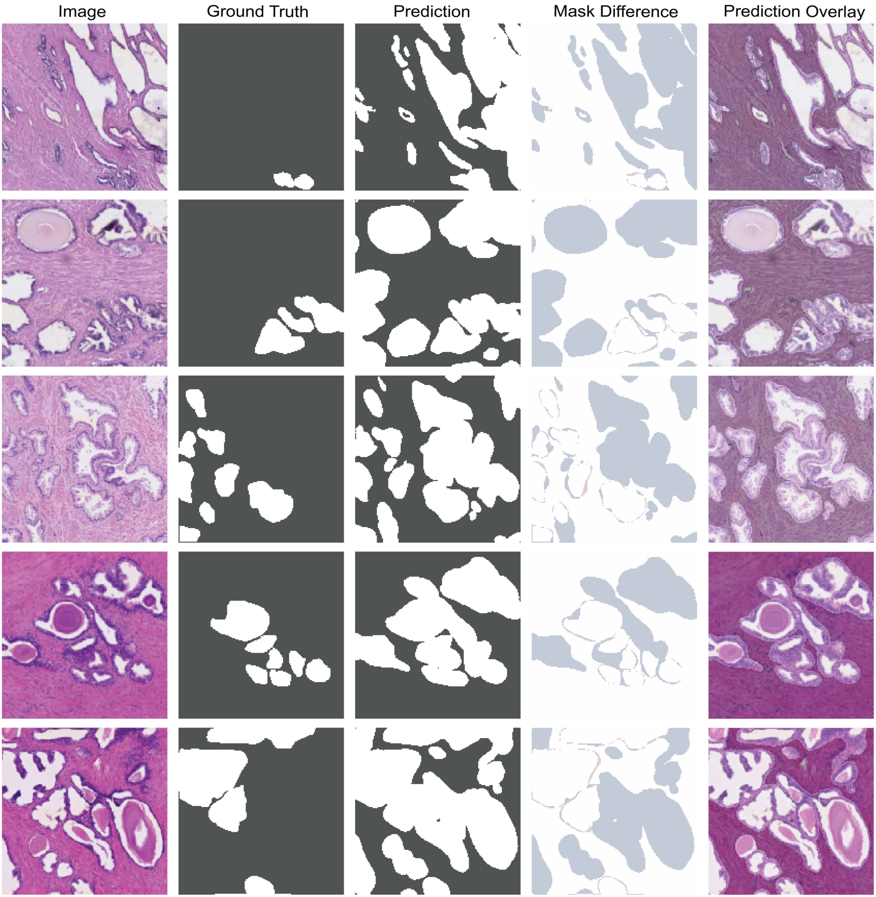
Image
Ground Truth
Prediction
Mask Difference
Prediction Overlay

Supplementary Figure 21. Best five predictions for spleen images from Team 2. Ground truth and predictions for Team 2's final solution are shown, along with the prediction overlay and mask difference with per-pixel false positives (in blue) and false negatives (red).

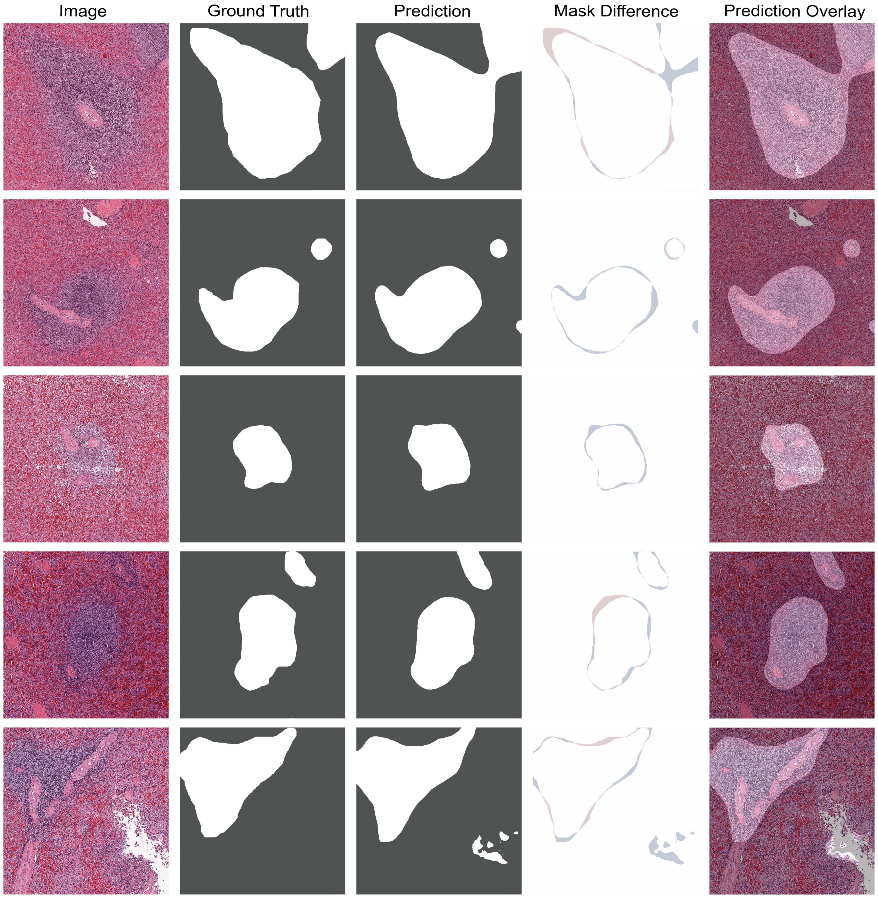
Image
Ground Truth
Prediction
Mask Difference
Prediction Overlay

Supplementary Figure 22. Worst five predictions for spleen images from Team 2. Ground truth and predictions for Team 2's final solution are shown, along with the prediction overlay and mask difference with per-pixel false positives (in blue) and false negatives (red).

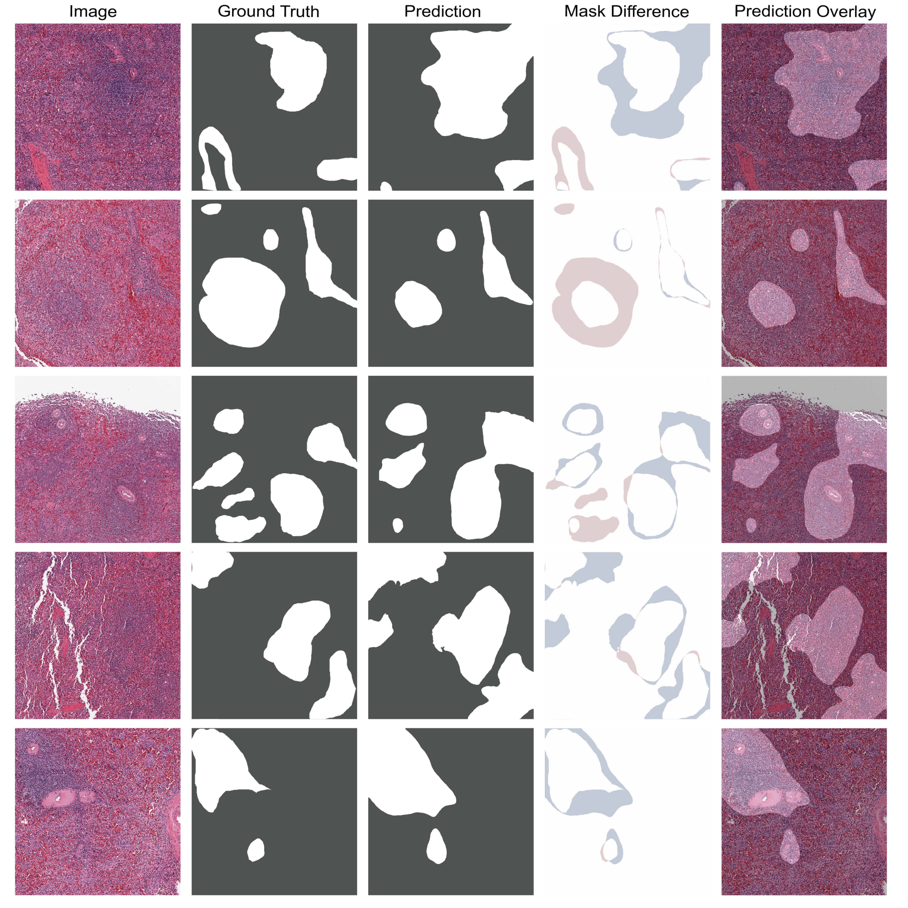
Image
Ground Truth
Prediction
Mask Difference
Prediction Overlay

Supplementary Figure 23. Best five predictions for kidney images from Team 3. Ground truth and predictions for Team 3's final solution are shown, along with the prediction overlay and mask difference with per-pixel false positives (in blue) and false negatives (red).

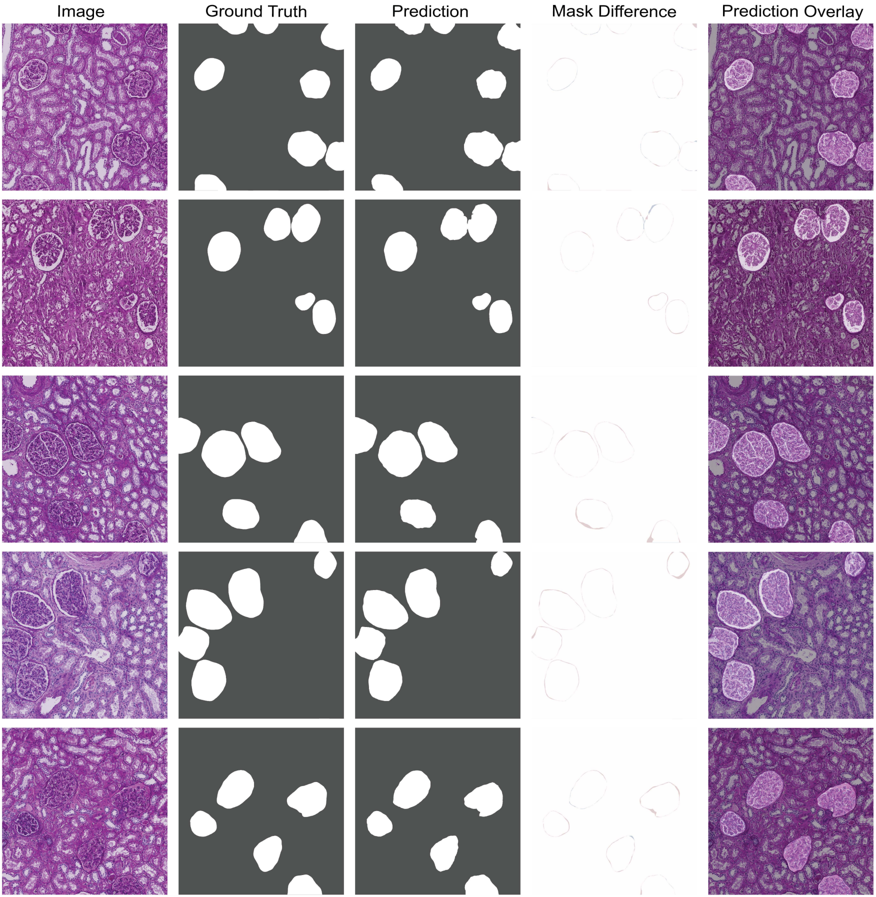
Image
Ground Truth
Prediction
Mask Difference
Prediction Overlay

Supplementary Figure 24. Worst five predictions for kidney images from Team 3. Ground truth and predictions for Team 3's final solution are shown, along with the prediction overlay and mask difference with per-pixel false positives (in blue) and false negatives (red).

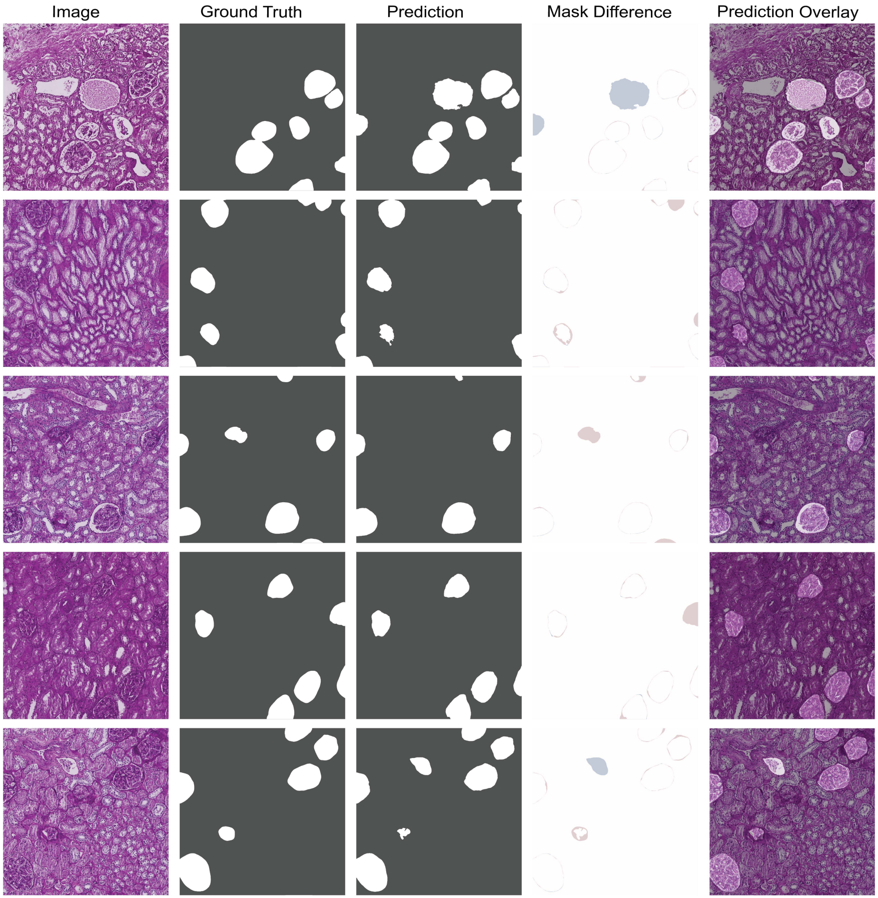
Image
Ground Truth
Prediction
Mask Difference
Prediction Overlay

Supplementary Figure 25. Best five predictions for large intestine images from Team 3. Ground truth and predictions for Team 3's final solution are shown, along with the prediction overlay and mask difference with per-pixel false positives (in blue) and false negatives (red).

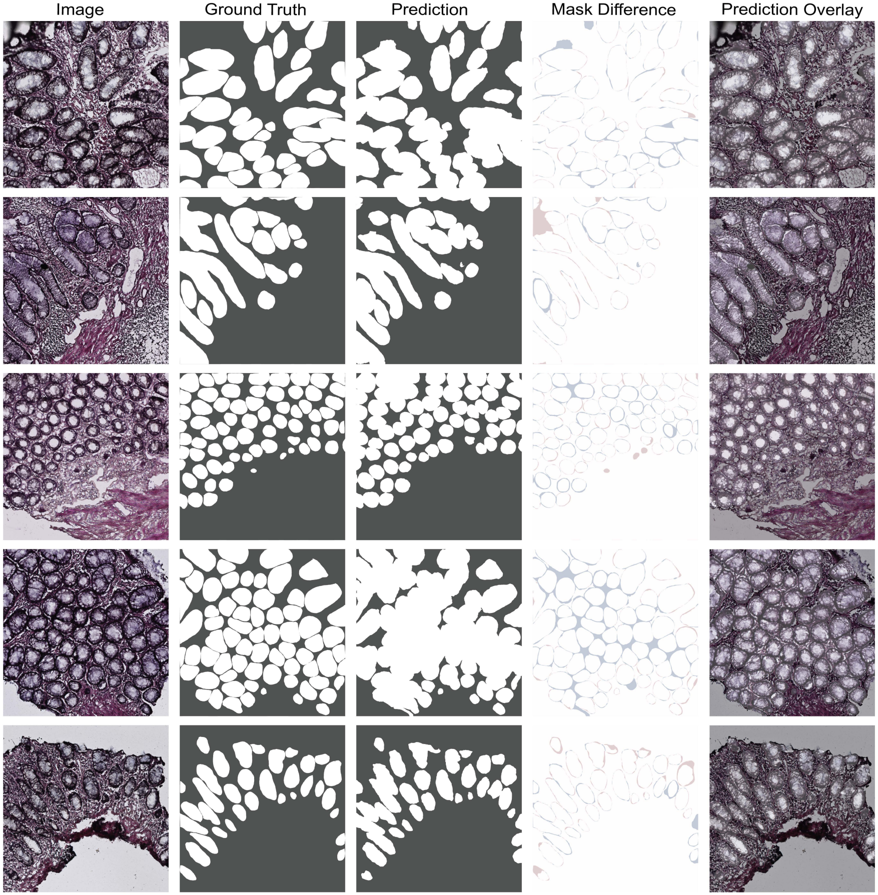
Image
Ground Truth
Prediction
Mask Difference
Prediction Overlay

Supplementary Figure 26. Worst five predictions for large intestine images from Team 3. Ground truth and predictions for Team 3's final solution are shown, along with the prediction overlay and mask difference with per-pixel false positives (in blue) and false negatives (red).

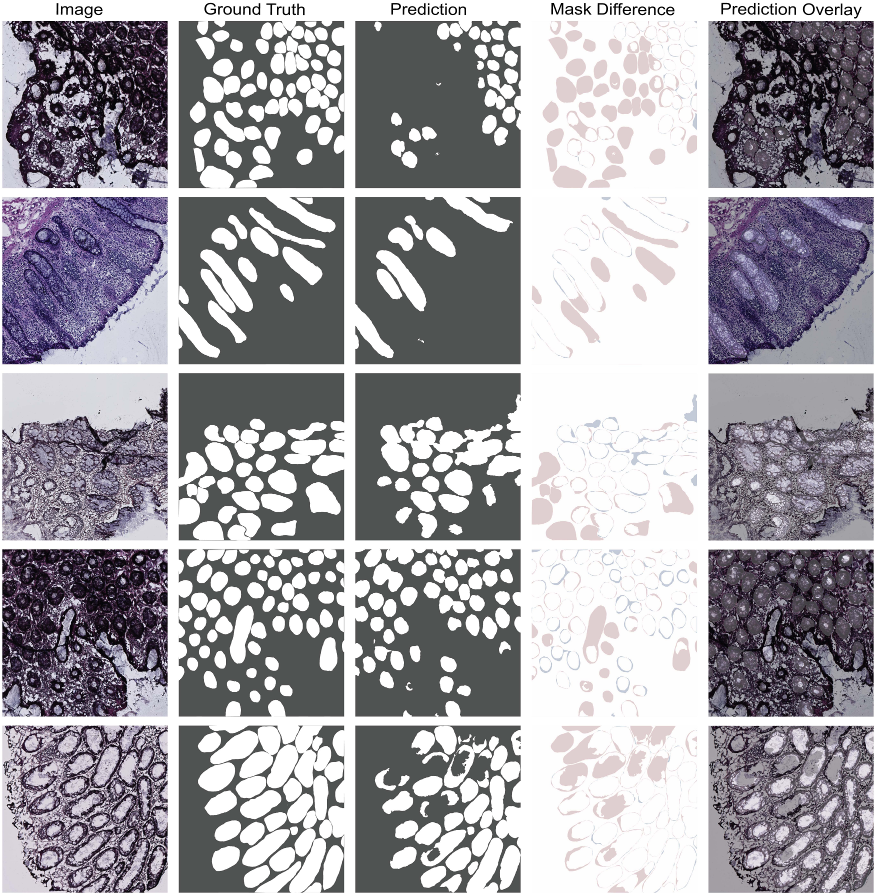
Image
Ground Truth
Prediction
Mask Difference
Prediction Overlay

Supplementary Figure 27. Best five predictions for lung images from Team 3. Ground truth and predictions for Team 3's final solution are shown, along with the prediction overlay and mask difference with per-pixel false positives (in blue) and false negatives (red).

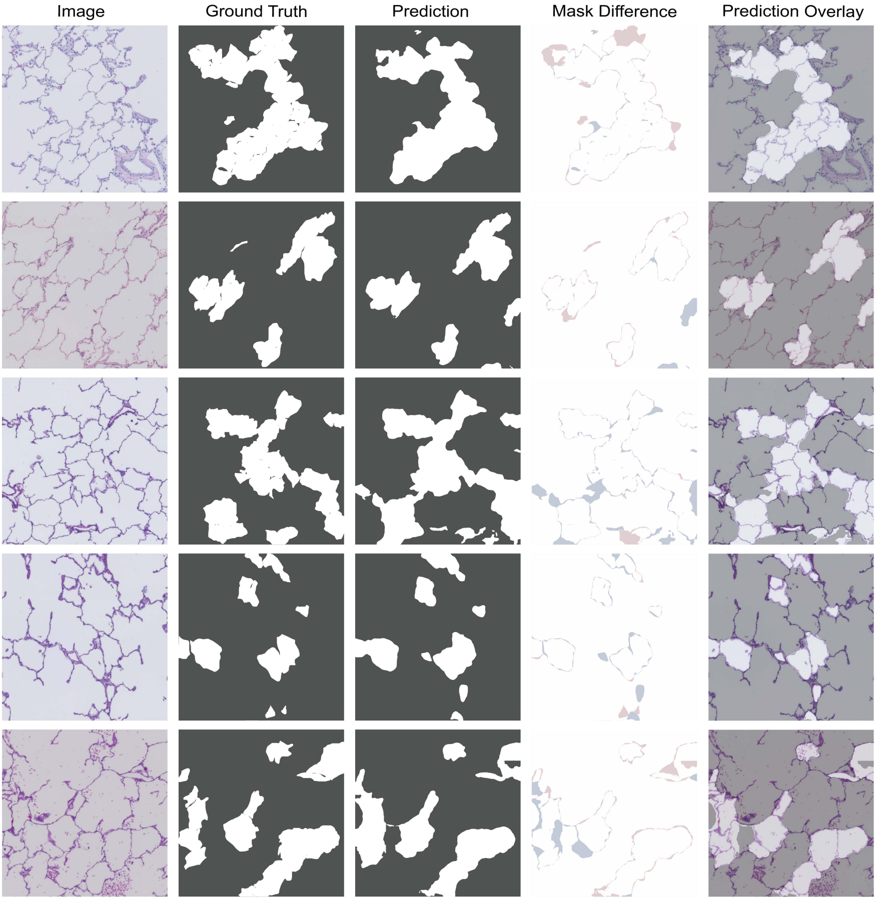
Image
Ground Truth
Prediction
Mask Difference
Prediction Overlay

Supplementary Figure 28. Worst five predictions for lung images from Team 3. Ground truth and predictions for Team 3's final solution are shown, along with the prediction overlay and mask difference with per-pixel false positives (in blue) and false negatives (red).

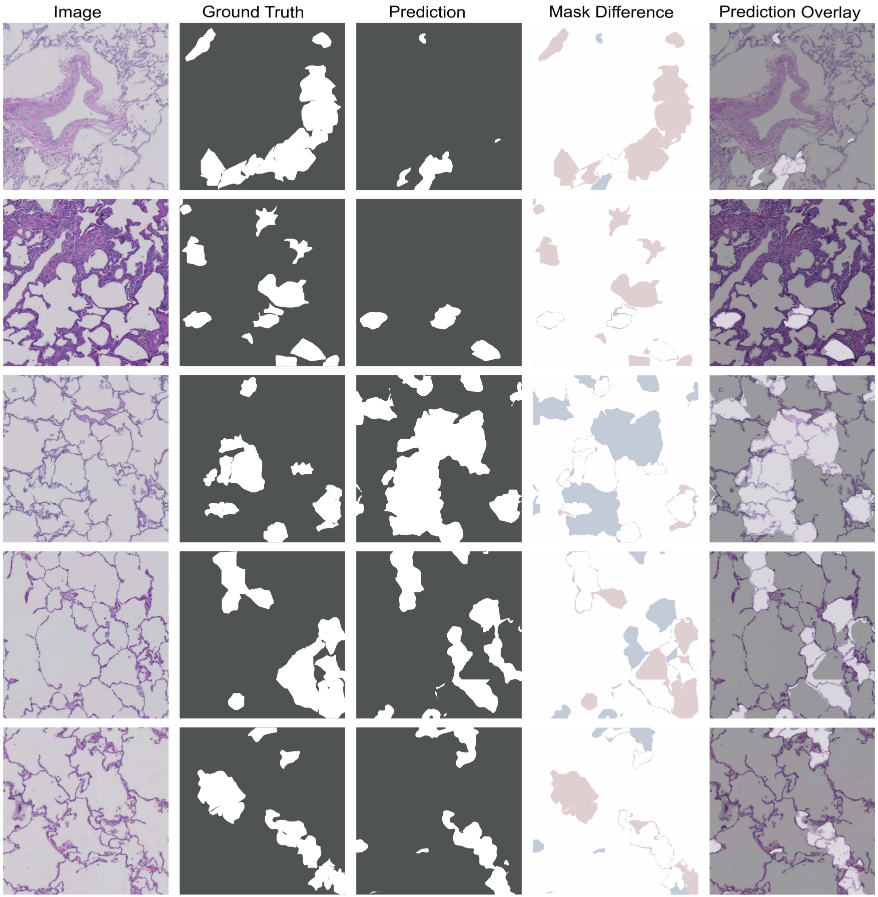
Image
Ground Truth
Prediction
Mask Difference
Prediction Overlay

Supplementary Figure 29. Best five predictions for prostate images from Team 3. Ground truth and predictions for Team 3's final solution are shown, along with the prediction overlay and mask difference with per-pixel false positives (in blue) and false negatives (red).

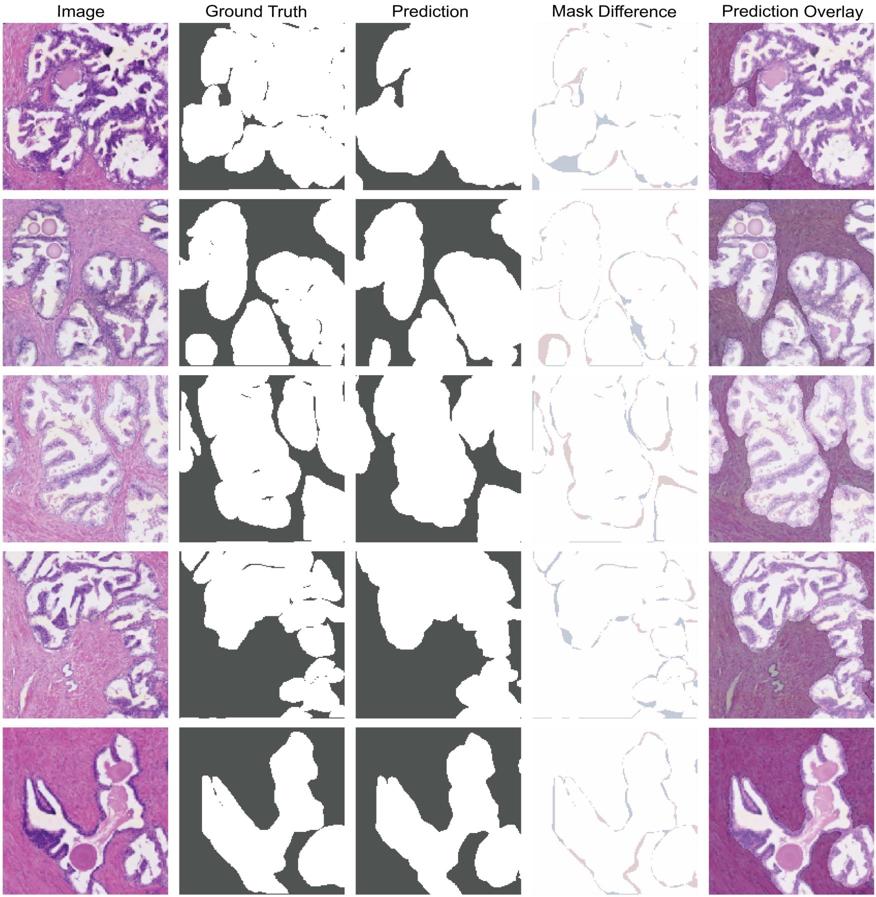
Image
Ground Truth
Prediction
Mask Difference
Prediction Overlay

Supplementary Figure 30. Worst five predictions for prostate images from Team 3. Ground truth and predictions for Team 3's final solution are shown, along with the prediction overlay and mask difference with per-pixel false positives (in blue) and false negatives (red).

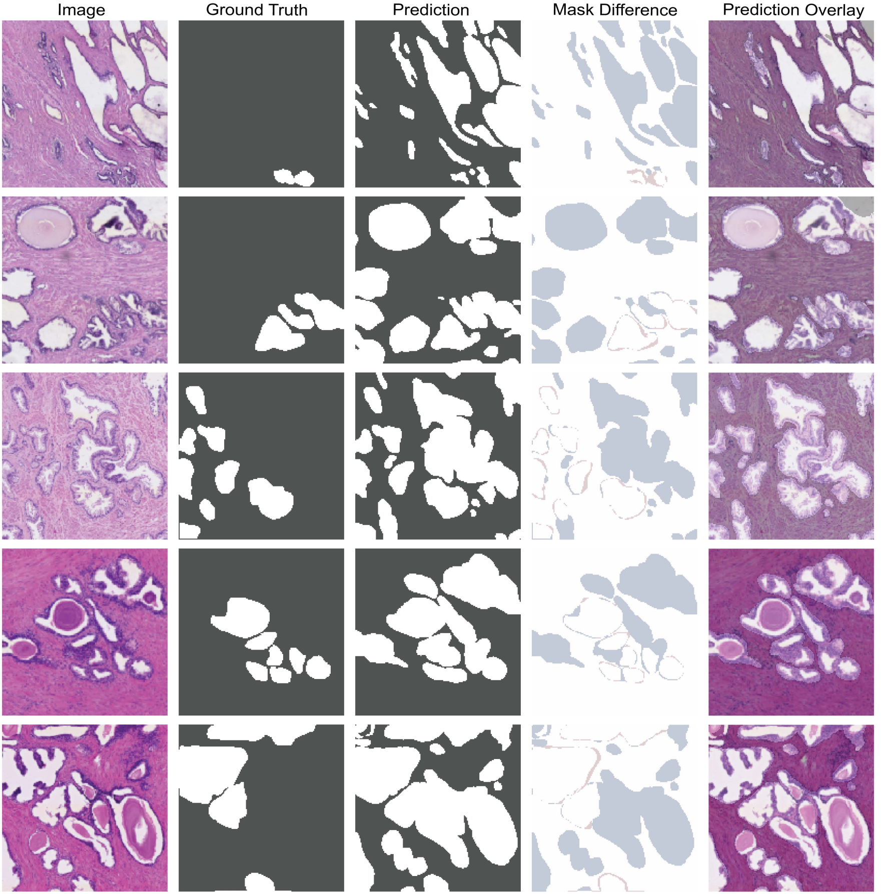
Image
Ground Truth
Prediction
Mask Difference
Prediction Overlay

Supplementary Figure 31. Best five predictions for spleen images from Team 3. Ground truth and predictions for Team 3's final solution are shown, along with the prediction overlay and mask difference with per-pixel false positives (in blue) and false negatives (red).

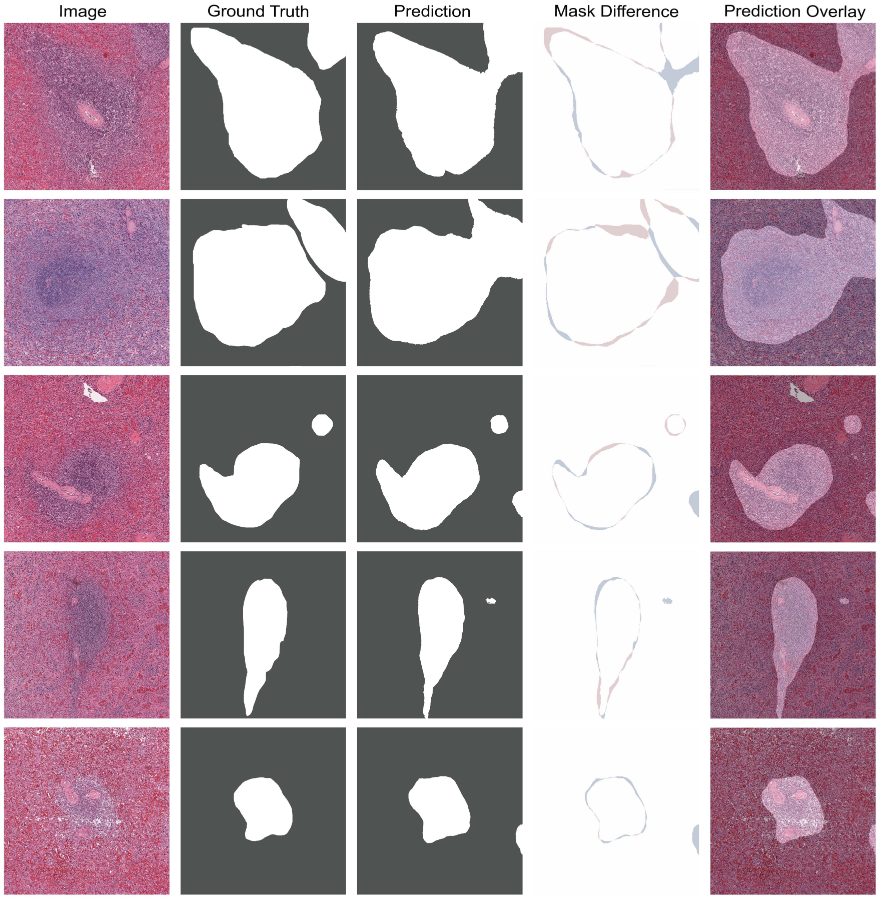
Image
Ground Truth
Prediction
Mask Difference
Prediction Overlay

Supplementary Figure 32. Worst five predictions for spleen images from Team 3. Ground truth and predictions for Team 3's final solution are shown, along with the prediction overlay and mask difference with per-pixel false positives (in blue) and false negatives (red).

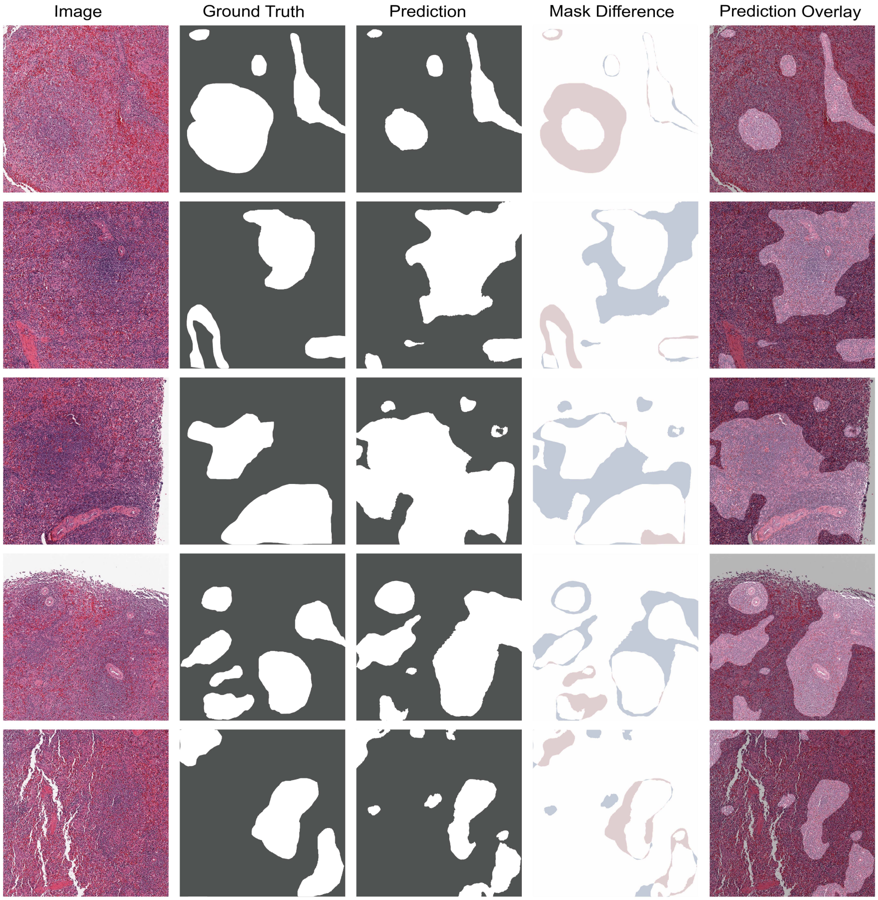
Image
Ground Truth
Prediction
Mask Difference
Prediction Overlay

Supplementary References

1. Xie, E. *et al.* SegFormer: Simple and Efficient Design for Semantic Segmentation with Transformers. in *Advances in Neural Information Processing Systems* vol. 34 12077–12090 (Curran Associates, Inc., 2021).
2. Cordts, M. *et al.* The cityscapes dataset for semantic urban scene understanding. in *Proceedings of the IEEE conference on computer vision and pattern recognition* 3213–3223 (2016).
3. Contributors, Mms. MMSegmentation: OpenMMLab Semantic Segmentation Toolbox and Benchmark. (2020).
4. Ruby, U. & Yendapalli, V. Binary cross entropy with deep learning technique for Image classification. *Int. J. Adv. Trends Comput. Sci. Eng.* **9**, (2020).
5. Yu, J. & Blaschko, M. The Lovász Hinge: A Convex Surrogate for Submodular Losses. 26.
6. Loshchilov, I. & Hutter, F. Decoupled Weight Decay Regularization. in *International Conference on Learning Representations* (2019).
7. Wightman, R. PyTorch Image Models. *GitHub repository* (2019) doi:10.5281/zenodo.4414861.
8. Tan, M. & Le, Q. V. EfficientNet: Rethinking Model Scaling for Convolutional Neural Networks. *ArXiv190511946 Cs Stat* (2020).
9. Liu, Z. *et al.* A ConvNet for the 2020s. Preprint at https://doi.org/10.48550/arXiv.2201.03545 (2022).
10. Tan, M. & Le, Q. V. EfficientNetV2: Smaller Models and Faster Training. Preprint at https://doi.org/10.48550/arXiv.2104.00298 (2021).
11. Xu, W., Xu, Y., Chang, T. & Tu, Z. Co-Scale Conv-Attentional Image Transformers. in *2021 IEEE/CVF International Conference on Computer Vision (ICCV)* 9961–9970 (2021). doi:10.1109/ICCV48922.2021.00983.

12. Liu, Z. *et al.* Swin Transformer: Hierarchical Vision Transformer using Shifted Windows. in *2021 IEEE/CVF International Conference on Computer Vision (ICCV)* 9992–10002 (2021). doi:10.1109/ICCV48922.2021.00986.

13. Lin, T.-Y., Goyal, P., Girshick, R., He, K. & Dollár, P. Focal Loss for Dense Object Detection. Preprint at https://doi.org/10.48550/arXiv.1708.02002 (2018).

14. Xie, X., Zhou, P., Li, H., Lin, Z. & Yan, S. Adan: Adaptive Nesterov Momentum Algorithm for Faster Optimizing Deep Models. Preprint at https://doi.org/10.48550/arXiv.2208.06677 (2023).

15. Howard, Y. J., Andy Lawrence, Bud Sims, Eddie Tinsley, Jarek Kazmierczak, Katy Börner, Leah Godwin, Marcos Novaes, Phil Culliton, Richard Holland, Rick Watson Addison. HuBMAP - Hacking the Kidney. (2020).

16. GeertLitjens, W. B., Hans Pinckaers, Kimmo Kartasalo, Maggie, Martin Eklund, PekkaRuusuvuori, PeterStröm, Sohier Dane. Prostate cANcer graDe Assessment (PANDA) Challenge. (2020).

17. Clark, K. *et al.* The Cancer Imaging Archive (TCIA): Maintaining and Operating a Public Information Repository. *J. Digit. Imaging* **26**, 1045–1057 (2013).

18. Byfield, P. Peter554/StainTools: Patch release for DOI. (2019) doi:10.5281/zenodo.3403170.

19. Vahadane, A. *et al.* Structure-Preserving Color Normalization and Sparse Stain Separation for Histological Images. *IEEE Trans. Med. Imaging* **35**, 1962–1971 (2016).

20. Sydorskyi, V., Krashenyi, I., Savka, D. & Zarichkovyi, O. Semi-Supervised Segmentation of Functional Tissue Units at the Cellular Level. Preprint at https://doi.org/10.48550/arXiv.2305.02148 (2023).

21. Zhou, Z., Rahman Siddiquee, M. M., Tajbakhsh, N. & Liang, J. UNet++: A Nested U-Net Architecture for Medical Image Segmentation. in *Deep Learning in Medical Image Analysis*

and Multimodal Learning for Clinical Decision Support (eds. Stoyanov, D. et al.) 3–11 (Springer International Publishing, 2018). doi:10.1007/978-3-030-00889-5_1.

22. Ronneberger, O., Fischer, P. & Brox, T. U-Net: Convolutional Networks for Biomedical Image Segmentation. in *Medical Image Computing and Computer-Assisted Intervention – MICCAI 2015* (eds. Navab, N., Hornegger, J., Wells, W. M. & Frangi, A. F.) 234–241 (Springer International Publishing, 2015). doi:10.1007/978-3-319-24574-4_28.
23. Chen, J.-N. *et al.* Transmix: Attend to mix for vision transformers. in *Proceedings of the IEEE/CVF Conference on Computer Vision and Pattern Recognition* 12135–12144 (2022).
24. Deng, J. *et al.* ImageNet: A Large-Scale Hierarchical Image Database. 8.
25. Kirillov, A., Wu, Y., He, K. & Girshick, R. Pointrend: Image segmentation as rendering. in *Proceedings of the IEEE/CVF conference on computer vision and pattern recognition* 9799–9808 (2020).
26. Iakubovskii, P. Segmentation Models Pytorch. *GitHub repository* (2019).
27. Kingma, D. P. & Ba, J. Adam: A Method for Stochastic Optimization. Preprint at https://doi.org/10.48550/arXiv.1412.6980 (2017).
28. Jaccard, P. The Distribution of the Flora in the Alpine Zone.1. *New Phytol.* **11**, 37–50 (1912).
29. Walt, S. van der *et al.* scikit-image: image processing in Python. *PeerJ* **2**, e453 (2014).
30. Yun, S. *et al.* CutMix: Regularization Strategy to Train Strong Classifiers With Localizable Features. in 6023–6032 (2019).
