# Supplementary material for: Segmenting functional tissue units across human organs using community-driven development of generalizable machine learning algorithms
Source: Nat Commun. 2023 Aug 3;14:4656. doi: 10.1038/s41467-023-40291-0 (PMC10400613; doi:10.1038/s41467-023-40291-0)
Supplement: Supplementary file 2 — Reporting Summary [file 41467_2023_40291_MOESM2_ESM.pdf]

Corresponding author(s): Katy Börner
Yashvardhan Jain

Last updated by author(s): Jul 14, 2023

Reporting Summary

Nature Portfolio wishes to improve the reproducibility of the work that we publish. This form provides structure for consistency and transparency in reporting. For further information on Nature Portfolio policies, see our Editorial Policies and the Editorial Policy Checklist.

Statistics

For all statistical analyses, confirm that the following items are present in the figure legend, table legend, main text, or Methods section.

n/a	Confirmed	
☐	☒	The exact sample size (*n*) for each experimental group/condition, given as a discrete number and unit of measurement
☒	☐	A statement on whether measurements were taken from distinct samples or whether the same sample was measured repeatedly
☐	☒	The statistical test(s) used AND whether they are one- or two-sided<br>*Only common tests should be described solely by name; describe more complex techniques in the Methods section.*
☒	☐	A description of all covariates tested
☒	☐	A description of any assumptions or corrections, such as tests of normality and adjustment for multiple comparisons
☐	☒	A full description of the statistical parameters including central tendency (e.g. means) or other basic estimates (e.g. regression coefficient) AND variation (e.g. standard deviation) or associated estimates of uncertainty (e.g. confidence intervals)
☐	☒	For null hypothesis testing, the test statistic (e.g. *F*, *t*, *r*) with confidence intervals, effect sizes, degrees of freedom and *P* value noted<br>*Give P values as exact values whenever suitable.*
☒	☐	For Bayesian analysis, information on the choice of priors and Markov chain Monte Carlo settings
☒	☐	For hierarchical and complex designs, identification of the appropriate level for tests and full reporting of outcomes
☒	☐	Estimates of effect sizes (e.g. Cohen's *d*, Pearson's *r*), indicating how they were calculated

Our web collection on statistics for biologists contains articles on many of the points above.

Software and code

Policy information about availability of computer code

Data collection	All code and relevant documentation used in this paper is available on GitHub at https://github.com/cns-iu/ccf-research-kaggle-2022. Microscopy image collection information (including antibody dilutions/amounts, validation, company names, catalog numbers and clone numbers for monoclonals) in previous publications and is not part of this work.
Data analysis	All code and relevant documentation used in this paper is available on GitHub at https://github.com/cns-iu/ccf-research-kaggle-2022. Additional major packages used are Python 3.9, Scipy 1.9.3, Scikit-learn 1.1.1, Matplotlib 3.5.1, Numpy 1.22.3, and Pandas 1.4.3.

For manuscripts utilizing custom algorithms or software that are central to the research but not yet described in published literature, software must be made available to editors and reviewers. We strongly encourage code deposition in a community repository (e.g. GitHub). See the Nature Portfolio guidelines for submitting code & software for further information.

Data

Policy information about availability of data

All manuscripts must include a data availability statement. This statement should provide the following information, where applicable:

- Accession codes, unique identifiers, or web links for publicly available datasets
- A description of any restrictions on data availability
- For clinical datasets or third party data, please ensure that the statement adheres to our policy

All curated data used in the competition (HuBMAP and HPA), along with the trained models from the winning teams, is publicly available via Zenodo. All primary competition data as well as external data used by Teams 1 and 2 is published on Zenodo at https://doi.org/10.5281/zenodo.7545744. All external data used by

Team 3 is available as Kaggle datasets, links to which are provided in the Supplementary Information. All trained model weights are published on Zenodo at https://doi.org/10.5281/zenodo.7545792. Source data for all plots presented in the paper are provided as a Zenodo dataset at https://doi.org/10.5281/zenodo.8144891.

Research involving human participants, their data, or biological material

Policy information about studies with human participants or human data. See also policy information about sex, gender (identity/presentation), and sexual orientation and race, ethnicity and racism.

Reporting on sex and gender	Data used in the study included metadata on sex of the human donors from who the tissue samples had been taken. This metadata was used for data sampling for building training and test sets for the machine learning models, with the aim of maintaining similar distributions of male and female donors across the dataset. All data used in this study is secondary data which was already collected as part of the HuBMAP and HPA projects. No study protocol was used as it was not applicable to this study.
Reporting on race, ethnicity, or other socially relevant groupings	N/A
Population characteristics	Age and sex metadata was used for data sampling for building datasets.
Recruitment	Data used consists of tissue samples from human donors as part of the HuBMAP and HPA efforts. All data used in this study is secondary data which was already collected as part of the HuBMAP and HPA projects. No study protocol was used as it was not applicable to this study.
Ethics oversight	All data used in this study is secondary data which was already collected as part of the HuBMAP and HPA projects. No study protocol was used as it was not applicable to this study.

Note that full information on the approval of the study protocol must also be provided in the manuscript.

Field-specific reporting

Please select the one below that is the best fit for your research. If you are not sure, read the appropriate sections before making your selection.

☒ Life sciences ☐ Behavioural & social sciences ☐ Ecological, evolutionary & environmental sciences

For a reference copy of the document with all sections, see nature.com/documents/nr-reporting-summary-flat.pdf

Life sciences study design

All studies must disclose on these points even when the disclosure is negative.

Sample size	The final curated dataset consisted of 880 images from both HuBMAP and HPA, with a total of 12,901 FTU instances annotated. The images were curated from a larger pool of data available from HuBMAP and HPA. The images were selected such that a balance can be maintained across male/female sex and across age groups. Images were selected from both HuBMAP and HPA data such that to maintain a balance between both sources. More implementation details are available in the Methods section in the manuscript.
Data exclusions	For the curation of the final dataset, images with damaged or unhealthy tissue were excluded. Images containing very low tissue region were also excluded, by calculating the tissue region percentage in the images using image processing. Specific implementation details are available in the Methods section in the manuscript.
Replication	All code submissions for inference were collected and graded automatically, which allows for reproduction of the scores. Final ranking was determined after re-running all team's chosen submissions. On competition end, all winning algorithms were validated and compared to scores on competition leaderboard. This was done once initially to validate the results and grant prizes to the top performing teams.
Randomization	The final curated dataset contained images from both HPA and HuBMAP data sources, to maintain variability. All HuBMAP data was kept for test sets and all publicly available HPA data was assigned to public training sets. The unpublished HPA data was assigned to the public test set. The assignment of data sources was intentional to maintain specific data sources across train/test sets but the assignment of individual images in the specific train/test sets was mostly random. The only criteria used was to balance donor sex and age across these datasets. Since the primary purpose of this curated dataset was to build machine learning models, the randomization provides the advantage of the algorithms not overfitting to human bias in sampling.
Blinding	The pathology experts that annotated the ground truth were aware of the specific tissue they were annotating but not necessarily aware of the donor metadata associated with it. The teams in the competition had access to the metadata associated with the public training data but did not have access to any information regarding the private test set (which was used for competition ranking and deciding winners) to prevent cheating in the competition and promote development of generalized algorithms.

Reporting for specific materials, systems and methods

We require information from authors about some types of materials, experimental systems and methods used in many studies. Here, indicate whether each material, system or method listed is relevant to your study. If you are not sure if a list item applies to your research, read the appropriate section before selecting a response.

Materials & experimental systems

n/a	Involved in the study
☒	☐ Antibodies
☒	☐ Eukaryotic cell lines
☒	☐ Palaeontology and archaeology
☒	☐ Animals and other organisms
☒	☐ Clinical data
☒	☐ Dual use research of concern
☒	☐ Plants

Methods

n/a	Involved in the study
☒	☐ ChIP-seq
☒	☐ Flow cytometry
☒	☐ MRI-based neuroimaging
